# VDJServer: A Cloud-Based Analysis Portal and Data Commons for Immune Repertoire Sequences and Rearrangements

**DOI:** 10.3389/fimmu.2018.00976

**Published:** 2018-05-08

**Authors:** Scott Christley, Walter Scarborough, Eddie Salinas, William H. Rounds, Inimary T. Toby, John M. Fonner, Mikhail K. Levin, Min Kim, Stephen A. Mock, Christopher Jordan, Jared Ostmeyer, Adam Buntzman, Florian Rubelt, Marco L. Davila, Nancy L. Monson, Richard H. Scheuermann, Lindsay G. Cowell

**Affiliations:** ^1^Department of Clinical Sciences, University of Texas Southwestern Medical Center, Dallas, TX, United States; ^2^Texas Advanced Computing Center, University of Texas at Austin, Austin, TX, United States; ^3^Bank of America Corporate Center, Charlotte, NC, United States; ^4^Bio5 Institute, University of Arizona, Tucson, AZ, United States; ^5^Department of Microbiology and Immunology, Institute for Immunity, Transplantation and Infection, Stanford University School of Medicine, Stanford, CA, United States; ^6^H. Lee Moffitt Cancer Center and Research Institute, Tampa, FL, United States; ^7^Department of Neurology and Neurotherapeutics, University of Texas Southwestern Medical Center, Dallas, TX, United States; ^8^Department of Immunology, University of Texas Southwestern Medical Center, Dallas, TX, United States; ^9^J. Craig Venter Institute, La Jolla, CA, United States; ^10^Department of Pathology, University of California, San Diego, San Diego, CA, United States; ^11^La Jolla Institute for Allergy & Immunology, La Jolla, CA, United States

**Keywords:** bioinformatics, cloud computing, Rep-seq, immune repertoire, B-cell receptor, T cell receptor

## Abstract

**Background:**

Recent technological advances in immune repertoire sequencing have created tremendous potential for advancing our understanding of adaptive immune response dynamics in various states of health and disease. Immune repertoire sequencing produces large, highly complex data sets, however, which require specialized methods and software tools for their effective analysis and interpretation.

**Results:**

VDJServer is a cloud-based analysis portal for immune repertoire sequence data that provide access to a suite of tools for a complete analysis workflow, including modules for preprocessing and quality control of sequence reads, V(D)J gene segment assignment, repertoire characterization, and repertoire comparison. VDJServer also provides sophisticated visualizations for exploratory analysis. It is accessible through a standard web browser *via* a graphical user interface designed for use by immunologists, clinicians, and bioinformatics researchers. VDJServer provides a data commons for public sharing of repertoire sequencing data, as well as private sharing of data between users. We describe the main functionality and architecture of VDJServer and demonstrate its capabilities with use cases from cancer immunology and autoimmunity.

**Conclusion:**

VDJServer provides a complete analysis suite for human and mouse T-cell and B-cell receptor repertoire sequencing data. The combination of its user-friendly interface and high-performance computing allows large immune repertoire sequencing projects to be analyzed with no programming or software installation required. VDJServer is a web-accessible cloud platform that provides access through a graphical user interface to a data management infrastructure, a collection of analysis tools covering all steps in an analysis, and an infrastructure for sharing data along with workflows, results, and computational provenance. VDJServer is a free, publicly available, and open-source licensed resource.

## Background

The adaptive immune system is composed of specialized cells, molecules, and processes that evolved to defend the organism against foreign pathogens and tumorous cells. In jawed vertebrates, the primary actors in adaptive immunity are B and T lymphocytes, which express immune receptors on their surface, and, in the case of B lymphocytes, secrete antibodies, a soluble form of the receptor. The genes encoding immune receptors are somatically generated through a DNA recombination process, V(D)J recombination, that assembles variable (V), diversity (D), and joining (J) gene segments into mature, composite genes (1). In some species, including mice and humans, the rearranged genes in B lymphocytes are further diversified through somatic hypermutation (SHM) (2). As a result of these processes, each individual has millions of unique immune receptor genes (3, 4), although some lymphocytes have identical genes due to clonal expansion. The full collection of functional immune receptor gene sequences in an individual at a single point in time is referred to as the adaptive immune receptor repertoire (AIRR). Somatic generation of a tremendously diverse repertoire enables effective immune responses against an essentially infinite array of antigens, such as those derived from pathogens or tumors. Components of this somatically generated repertoire can also recognize self-antigens, however, leading to autoimmune responses.

**Figure 1:**
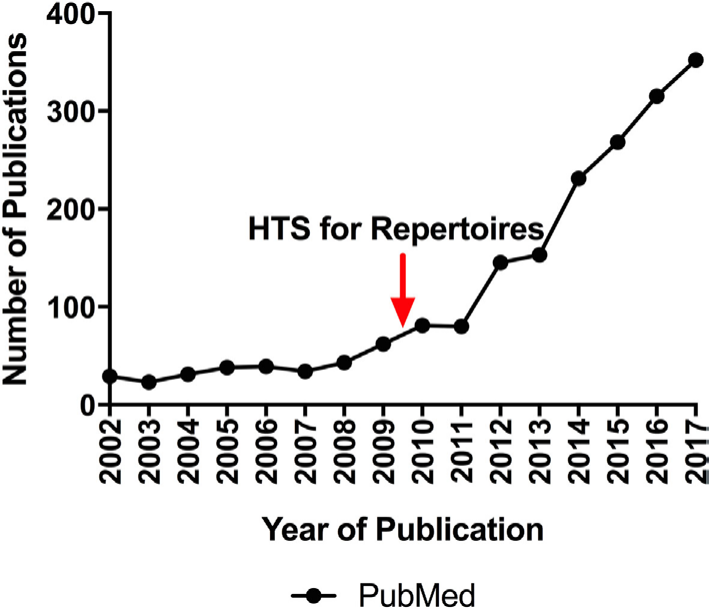
The number of newly published repertoire sequencing papers appearing in PubMed each year over the last 15 years. For 2002, the number was obtained by the query ((repertoire sequencing) AND (2002 [dp])). The number for 2017 is a projection based on the number appearing between January and December 2017. The red arrow indicates the year in which publications demonstrated the feasibility of applying high-throughput sequencing to immune repertoires.

The composition of immune repertoires shifts in response to immunological events (5). Thus, immune repertoires reflect the history and current state of adaptive immune responses, and analysis of repertoire composition is critical in both basic and translational research, clinical diagnostics, and in pharmaceutical development. Recent technological advances in immune repertoire sequencing have created tremendous potential for it to advance our understanding of adaptive immune response dynamics and lead to the development of repertoire-based diagnostic and prognostic assays. This has resulted in an explosion of research activity, a trend expected to continue (Figure 1). Recent examples of the power of repertoire analysis to have significant impact across various research and clinical areas include: understanding the development of a healthy immune system and immune senescence (6, 7); determining the nature of successful and unsuccessful immune responses for vaccine design (8–13); identifying the targets of autoimmune responses (14–18); diagnosing and monitoring hematologic malignancies (19–23); predicting clinical outcomes in cancer patients (24–30); and monitoring patients for graft-versus-host disease (31) or for organ rejection (32–34) after transplantation.

Immune repertoire sequencing produces large, highly complex data sets that require specialized analysis methods and software tools. Since the first studies demonstrating the technology were published (35–39), a research community has arisen around the development of new methods and tools (40, 41). As part of that effort, we developed VDJServer to address critical barriers in broader adoption of immune repertoire sequencing, namely, the lack of a complete, start-to-finish analysis pipeline, the lack of a data management infrastructure, and limited access for many researchers to high-performance computing (HPC) resources. VDJServer fills these gaps, specifically providing (1) an open suite of interoperable repertoire analysis tools that allows users to upload a set of sequences and pass them through a seamless workflow that executes all steps in an analysis, (2) access to sophisticated analysis tools running in an HPC environment, (3) interactive visualization capabilities for exploratory analysis, (4) a data management infrastructure, and (5) a graphical user interface to facilitate use by experimental and clinical research groups that lack bioinformatics expertise. VDJServer provides these capabilities as a free and publicly available resource.

While most immune repertoire analysis tools must be locally installed, there are a few tools that can be accessed over the web. The National Center for Biotechnology Information provides web access to the IgBlast tool for germline gene assignment (42). The Immunogenetics Information System (IMGT) provides access to tools for germline gene assignment (IMGT/V-QUEST) and junction analysis (IMGT/JunctionAnalysis) (43–47). The Vidjil web application (48) provides access to the Vidjil clonotype clustering algorithm, as well as to paired-end read merging *via* PEAR (49), and to germline gene alignment and clone identification using MiXCR (50). The most complete web-based analysis pipeline is provided by IGGalaxy (51) and its successor ARGalaxy (52). These are Galaxy-based (53) pipelines that provide access to demultiplexing and read trimming for 454 data, to downstream analysis tools, such as Change-O (54) and BASELINe (55), and to visualization of the output of those tools. ImmuneDB (56), which must be installed locally, provides a web-based interface to explore results from its analysis pipeline, which includes preprocessing with pRESTO (57), gene and clonal assignment (58), lineage tree construction, and mutation analysis with BASELINe (55). All of these web-based tools are limited in some fashion, however, either by restricting the number of sequences accepted by the web application, providing only a single tool suite, or not providing the tools necessary for all steps in a complete analysis workflow. Furthermore, none of these tools provide an HPC implementation to handle large immune repertoire studies, they lack metadata capabilities with user-defined sample groups and associated repertoire comparative analysis between groups, and they do not capture the necessary provenance information to allow for reproducibility of the analysis by others (59, 60). Among all currently available tools, VDJServer is the only web-accessible cloud platform that provides access through a graphical user interface to a data management infrastructure, a collection of HPC-enabled analysis tools covering all steps in an analysis, and an infrastructure for sharing data along with workflows, results, and computational provenance.

## Implementation

### Cloud-Based Architecture Overview

The VDJServer analysis portal is comprised of two main components: a web browser user interface and a web API. VDJServer’s architecture is designed upon the Agave Science-As-A-Service cloud platform (61) and augmented with a VDJServer-specific API. Generally, science gateways need to implement a database resource within their architecture for data management. However, the use of Agave allows VDJServer to offload database implementation into the cloud platform. This simplifies VDJServer’s architecture and provides the many benefits of cloud computing, such as lower maintenance costs, quick and flexible deployment, and dynamic scaling to accommodate user load. Agave Science APIs are a collection of RESTful web services with user identity management, file management, systems management, application deployment, metadata database, events/notifications, and job execution as some of their main functionality. VDJServer provides an additional RESTful API (Table 1) for project management, Agave event/notification processing, metadata capture for files and jobs, user profile and feedback management, community data publishing, and error logging. The API is implemented as a JavaScript Node.js application using the Express framework, and Nginx is the web server acting as HTTP/HTTPS proxy and serving the user interface code to client browsers.

**Table 1:** VDJServer release 1.0 API.

Endpoint	Method	Description
/	GET	Status of API service
/feedback	POST	User feedback
/feedback/public	POST	Public feedback
/jobs/queue/pending	GET	Pending jobs for project
/jobs/queue	POST	Submit job
/jobs/archive/:id	POST	Archive job
/jobs/unarchive/:id	POST	Unarchive job
/notifications/files/import	POST	Agave notifications for file import
/notifications/jobs/:id	POST	Agave notifications for job
/permissions/metadata	POST	Update user permissions for metadata
/permissions/username	POST	Add user permissions on project data
/permissions/username	DELETE	Remove user permissions from project data
/projects	POST	Create project
/projects/:id/metadata/export	GET	Export metadata from project into tab-separated values file
/projects/:id/metadata/import	POST	Import metadata into project
/public	GET	Query public data
/telemetry	POST	Error logging
/token	POST	Request an Agave authentication token
/token	PUT	Refresh Agave authentication token
/user	POST	Create user account
/user/change-password	POST	User change password
/user/reset-password	POST	Initiate password reset
/user/reset-password/verify	POST	Verify password reset
/user/:username/verify/email	POST	Send user verification email
/user/verify/:id	POST	Verify user

### Web Browser Interface

VDJServer’s user interface is a JavaScript single-page application with Backbone.js for the model-view event-driven application framework, Bootstrap for CSS web layout, and Handlebars for HTML templates. Data transport uses JSON with jQuery for some HTTP requests not handled by Backbone.js and Websockets for server-side events, such as file upload and job notifications. Chart and graph visualizations use D3.js, NVD3, and Highcharts. RequireJS is used for file and module loading optimization, and Grunt is used for the build system.

The interface has four primary views: project data management, metadata entry, configuration and execution of analysis jobs, and visualization of results. Each of these primary views is described in more detail in the following sections.

### Project Data Management

**Figure 2:**
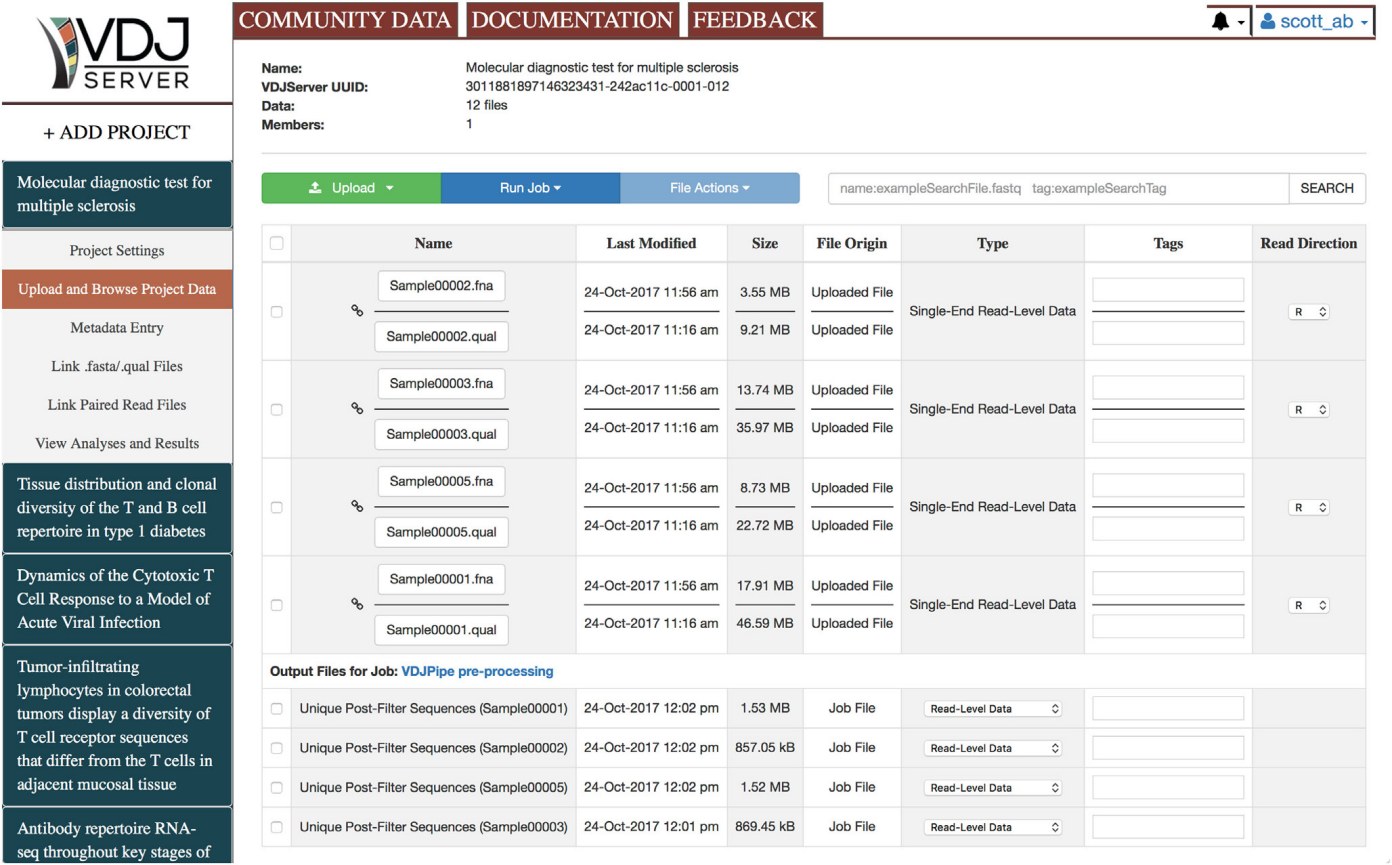
Web browser interface for Project Data Management. Along the top of the screen are tabs for Community Data, which displays public projects, Documentation, which opens up the documentation webpage in a new browser window, and Feedback, which allows you to send a message or ask a question. The left panel provides the list of user projects and the button to add a new project. When a project is selected, a submenu is shown to navigate the primary views: Project Settings, Upload and Browse Project Data, Metadata Entry, Link.fasta/.qual Files, Link Paired Read Files, and View Analyses and Results. The current view shown is Upload and Browse Project Data, which shows the list of user-uploaded files and some output files from a VDJPipe preprocessing job. The top of the view has project information, buttons for uploading files and running analysis jobs, and a search window to narrow the list of files displayed.

VDJServer is project-based where a project typically corresponds to a single experimental study. Each project is a logical container for files, jobs, analysis results, and visualizations, and any number of projects may be created. Figure 2 shows the interface for an example project. Multiple users can be given permissions on a project allowing them to run analysis jobs, access data, and visualize results that are shared with the other users. There is no limit to the number of files, jobs, or users that can be associated with a project. Data files can be uploaded from the user’s computer, from the user’s Dropbox account, or from a URL (HTTP and FTP supported protocols). VDJServer supports sequencing data in a number of file formats including single-end and paired-end reads in FASTQ format, single-end reads and quality scores in separate FASTA and QUAL files, and sequence data without quality scores in FASTA format. They can be compressed as zip, gzip, or bzip2 for faster upload. Each data file can be tagged with a pre-defined semantic type and with a set of user-defined tags. The semantic type allows VDJServer to automatically infer the appropriate matching between files and tool inputs for analysis jobs and to populate user interface elements with appropriate options. Both help to prevent analysis and job errors. The file search interface allows users to query all files within a project using a user-specified search string that searches against filenames, semantic type, and user-defined tags. Files are the underlying basis for input and output data for tools, and the resulting explosion in the number, type, and size of files can easily overwhelm users. VDJServer encapsulates this complexity and streamlines processing by displaying abbreviated summaries of job output, which is broadly grouped as output data (which may be input to another tool), visualization data, or log/configuration information. Context-specific operations appropriate for each output file type are provided, e.g., figures and charts are shown for visualization data. Regardless, the underlying files are always available to the user to download if desired.

### Study Metadata

Structured metadata are increasingly important to insure adherence to funder and journal data sharing policies for data reusability and study reproducibility. VDJServer provides a comprehensive metadata entry and management interface for an immune repertoire study and its subjects, samples, and biomaterial processing. VDJServer collects metadata according to the recently published Minimal Information about Adaptive Immune Receptor Repertoires (MiAIRR) standards developed by a working group of the AIRR Community^1^ (40, 41), as well as any number of user-defined fields. Custom groups of samples and subjects, e.g., Control and Treatment groups, can be defined by the user as part of the metadata. VDJServer uses metadata for performing immune repertoire calculations and comparisons between samples and groups. Metadata can be provided through manual entry, uploading of a spreadsheet table, or a combination of the two. Metadata can be exported into Tab-Separated Values (TSV) files for easy import into external tools, such as Microsoft Excel. VDJServer automatically captures provenance metadata for all analysis workflows executed by the users, which is described in more detail in Section “Findable, Accessible, Interoperable, and Reusable (FAIR) Principles and Reproducibility.”

### Analysis Workflows

**Figure 3:**
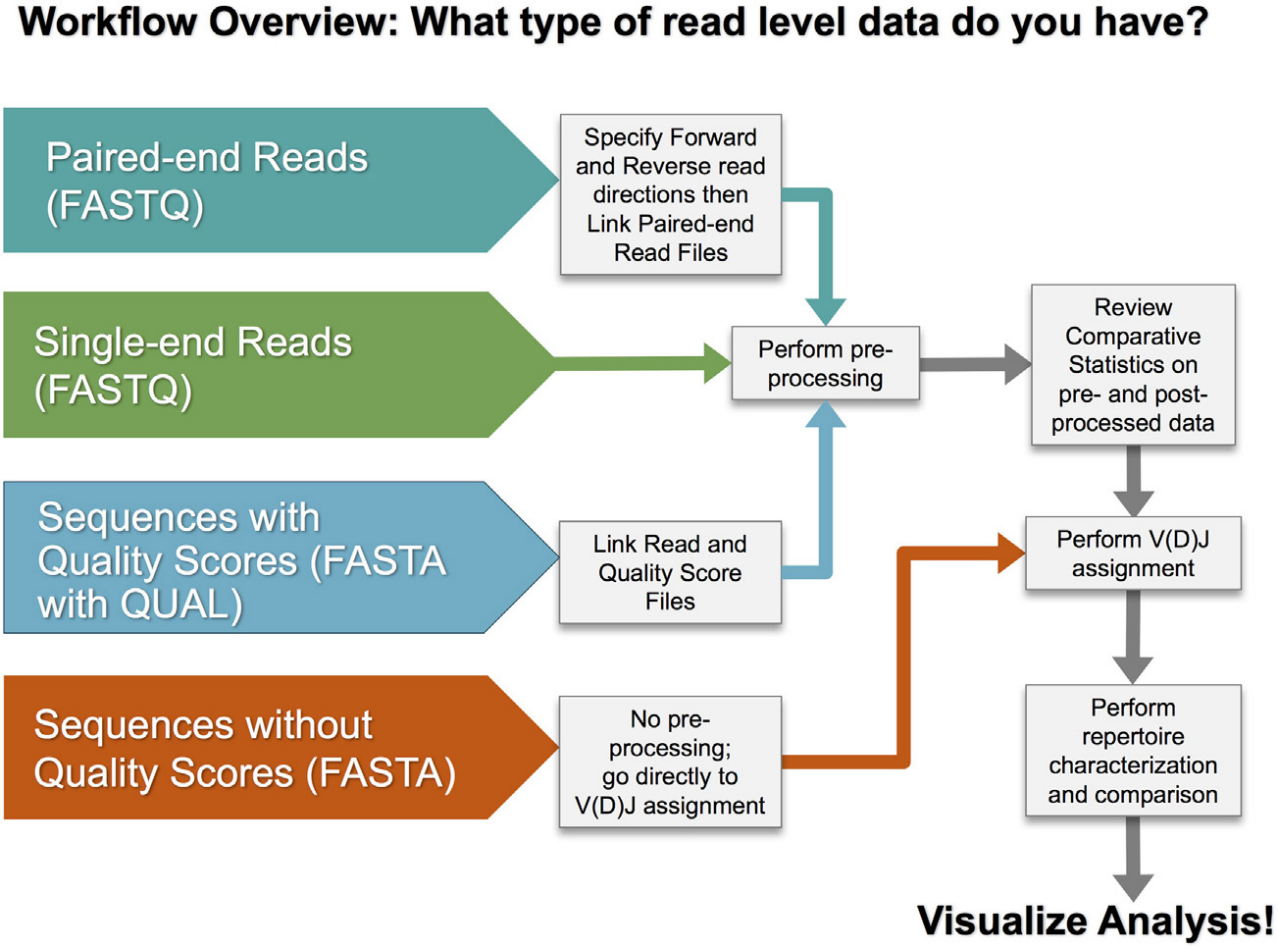
VDJServer analysis workflow overview.

There are multiple steps in a typical analysis workflow for repertoire sequencing data, as illustrated in Figure 3. The four basic steps include (1) preprocessing of sequence reads, (2) V(D)J gene segment assignment, (3) rearrangement annotation, and (4) repertoire characterization and comparison. VDJServer provides tools for all steps consisting of VDJPipe (62) and pRESTO (57) for step 1, IgBlast (42) for step 2, RepSum and Change-O (54) for step 3, and RepCalc, Change-O (54), Alakazam (54), and SHazaM (54) for step 4. Not all steps are required, and specific workflows will vary based upon the input sequencing data and desired analysis.

Preprocessing of sequence reads is a common step for next-generation sequencing data that remove low quality reads and prepares the reads for further analysis. VDJServer provides preprocessing tools designed specifically for immune repertoire sequencing data, since preprocessing of these data has unique characteristics, such as 5′ and 3′ PCR primer targeting, complex multilevel barcode demultiplexing, and duplicate sequence read collapsing, when compared with other next-generation sequencing data.^2^^,^^3^ Preprocessing tasks may include quality filtering, homopolymer filtering, length and nucleotide filtering, merging of paired-end reads, barcode demultiplexing, forward and reverse primer matching, and duplicate reads collapsing. Users can choose either or both VDJPipe and pRESTO for preprocessing as they are similar in capability but each with some unique features (e.g., pRESTO supports preprocessing of reads containing unique molecular identifiers). VDJServer calculates base composition statistics and read quality statistics before and after preprocessing and provides comparative visualization for user assessment, as described in Section “Visualizations.”

VDJServer combines the V(D)J gene segment assignment and rearrangement annotation steps together in a single job execution, and there is no restriction on the number of sequences that may be submitted. IgBlast is used for V(D)J gene segment assignment. The germline database used by VDJServer contains T cell and B cell gene sequences for human and mouse based upon the IMGT database (63). Multiple annotation outputs are provided by VDJServer including: a VDJML (64) file of the IgBlast output alignments, a TSV file provided by RepSum, a TSV file provided by Change-O, and an AIRR rearrangements file. The TSV files contain annotations, such as gene calls and CDR3 sequences. The AIRR rearrangements file is an evolving community standard to facilitate interoperability between immune repertoire analysis tools,^4^ and it specifies mandatory and optional annotations. Mandatory annotations are all essential data fields from V(D)J gene segment assignment, such as the V, D, and J gene calls, functional versus non-functional rearrangements, CIGAR strings for sequence alignment, and sequences and their identifiers. Optional annotations are those which can be computed from the mandatory annotations or are specific to some V(D)J gene segment assignment tools.

After the initial processing steps to assemble individual VDJ rearrangements, repertoire characterization and comparison becomes the primary focus of the researcher to extract insights and test hypotheses with their data. VDJServer provides a wide range of functionality applicable to both T-cell and B-cell data. Repertoire characterization encompasses various factors for each repertoire sample, including (1) enumeration of V, D, and J gene segment usage, and V–J and V–D–J combinations; (2) identification of clonally related sequences; (3) estimation of repertoire diversity utilizing common measures of diversity; (4) characterization of CDR3 patterns, such as length, amino acid utilization, and physicochemical properties; and (5) enumeration of unique CDR3 sequences and unique V gene, J gene, and CDR3 sequence combinations. VDJServer also provides B-cell-specific functionality for analysis of SHM: (1) characterization of somatic mutation patterns, including identification of mutations, determination of replacement (R) or silent (S) mutations, calculation of V gene, CDR, and framework mutation frequencies and R:S ratios; (2) characterization of patterns of selection across framework and CDR regions; and (3) inference of B cell lineage trees. VDJServer provides comparison of many of these characteristics between repertoire samples and repertoire groups. As described earlier in Section “Study Metadata,” users can define their own groupings of repertoire samples, which will be pairwise compared and provides increased flexibility for *ad hoc* analysis. How group comparisons are performed depends upon the nature of the characteristic. For numerical values, such as gene segment usage, mean and variance are calculated for the set of repertoires that comprise the group. Not all characteristics, such as a diversity curve, have a well-established aggregation metric and thus do not have a meaningful group comparison. While other characteristics enable additional analyses, such as shared CDR3 sequences, with intragroup comparison quantifying sharing between repertoires within the same group and intergroup comparison quantifying sharing between two groups. Results from repertoire characterization and comparison can be visually examined through a set of charts and figures, as described in the next section. Furthermore, all of the results are stored in TSV files that can be downloaded for import into external tools.

### Visualizations

VDJServer provides two primary sets of visualizations. One set of charts for assessing quality and composition statistics before and after preprocessing of sequence reads, and another set of charts for examining repertoire characteristics and comparative repertoire analysis. All charts provide interactive settings that allow the user to manipulate the chart and enable/disable which results are displayed, while tooltips (a small visual popup box) provide additional detail for specific data points when the mouse pointer hovers over them. In addition, the currently displayed chart can be quickly downloaded as a high-resolution image with the click of a button.

**Figure 4:**
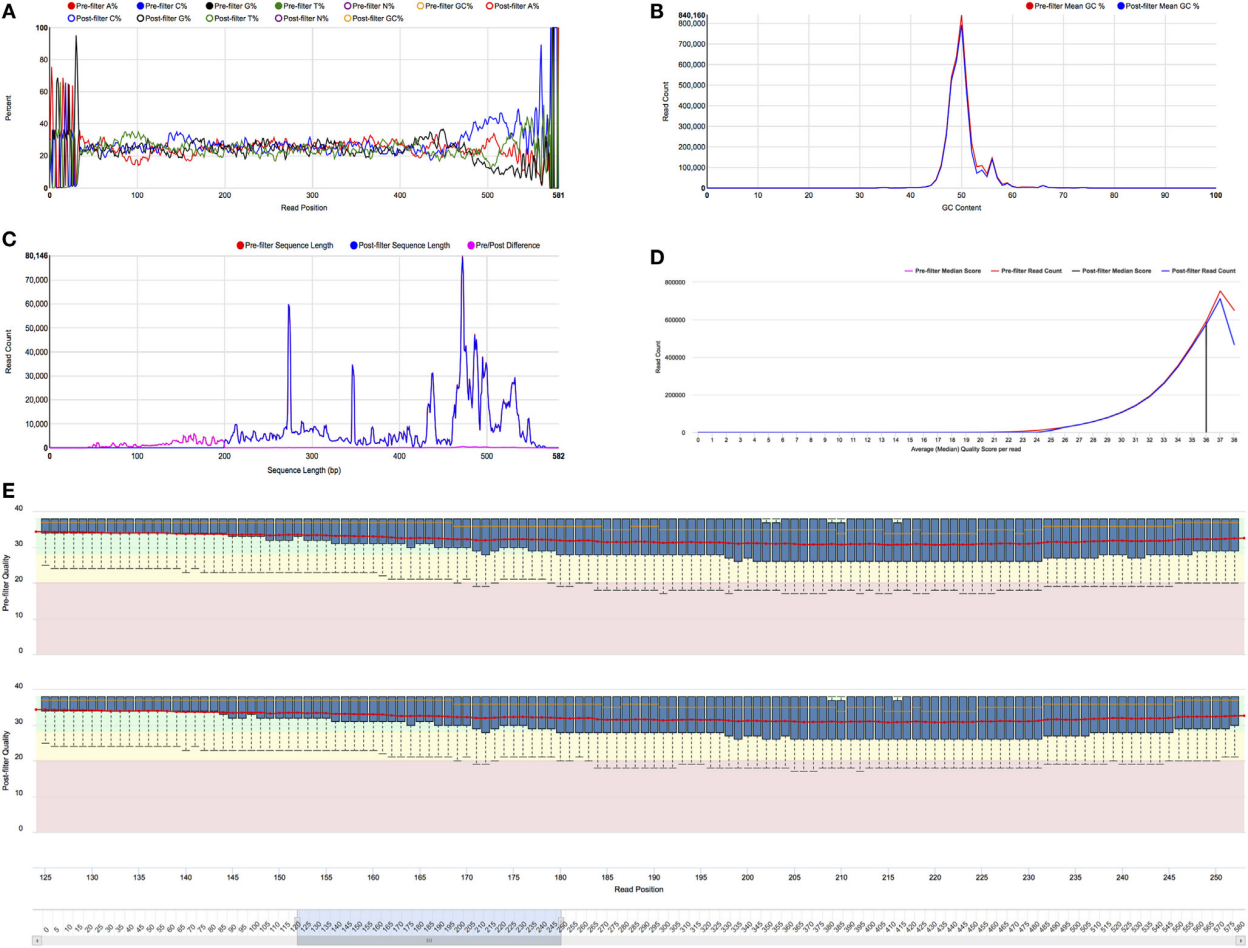
Preprocessing visualizations. All of the charts provide pre- and post-filtering statistics for side-by-side comparison. Full size versions of the figures are provided in Supplementary Material. **(A)** Nucleotide composition for each read position. Each colored line represents the percentage of a nucleotide or ambiguous base call (N) at each read position. The legend functions as a toggle to set which lines are shown. This figure is currently showing the composition pre-filtering for A, C, G, and T nucleotides. **(B)** GC content histogram. The graph shows the number of reads along the *Y* axis and GC percentage along the *X* axis. The red curve indicates the number for pre-filter reads, and the blue curve represents the number for post-filter reads. **(C)** Sequence length histogram. The graph shows the number of reads along the *Y* axis and sequence length along the *X* axis. The red curve shows the number of sequences of each length for pre-filter reads, the blue curve shows the number for post-filter reads, and the magenta curve shows the pre/post difference. The graph clearly shows that a length filter of 200 was used. **(D)** Mean quality score histogram. The graph shows the number of reads along the *Y* axis and the average quality score along the *X* axis. The red curve shows the number of sequences for pre-filter reads, and the blue curve shows the number for post-filter reads. The magenta and black vertical lines show the median score (36) for the pre- and post-filter reads, respectively. **(E)** Quality score distribution for each read position. Quality score is shown on the *Y* axis, and read position is shown on the *X* axis. At each position, the box-and-whiskers plot shows the median quality score, inter-quartile range, and the 10th and 90th quantiles. The *X* axis has zoom control, which is set to positions 125–250 in this figure.

The preprocessing visualizations include histograms of read length, average quality score, and GC content; a box-and-whiskers display of the quality score distribution at each read position; and base composition at each read position for all four bases and ambiguous base calls. The quality score distribution has the ability to zoom in on portions of sequence read. Statistics are calculated on the pre- and postprocessed sequence reads, and both are provided on a single figure for easy comparison. Examples of these charts are shown in Figure 4.

**Figure 5:**
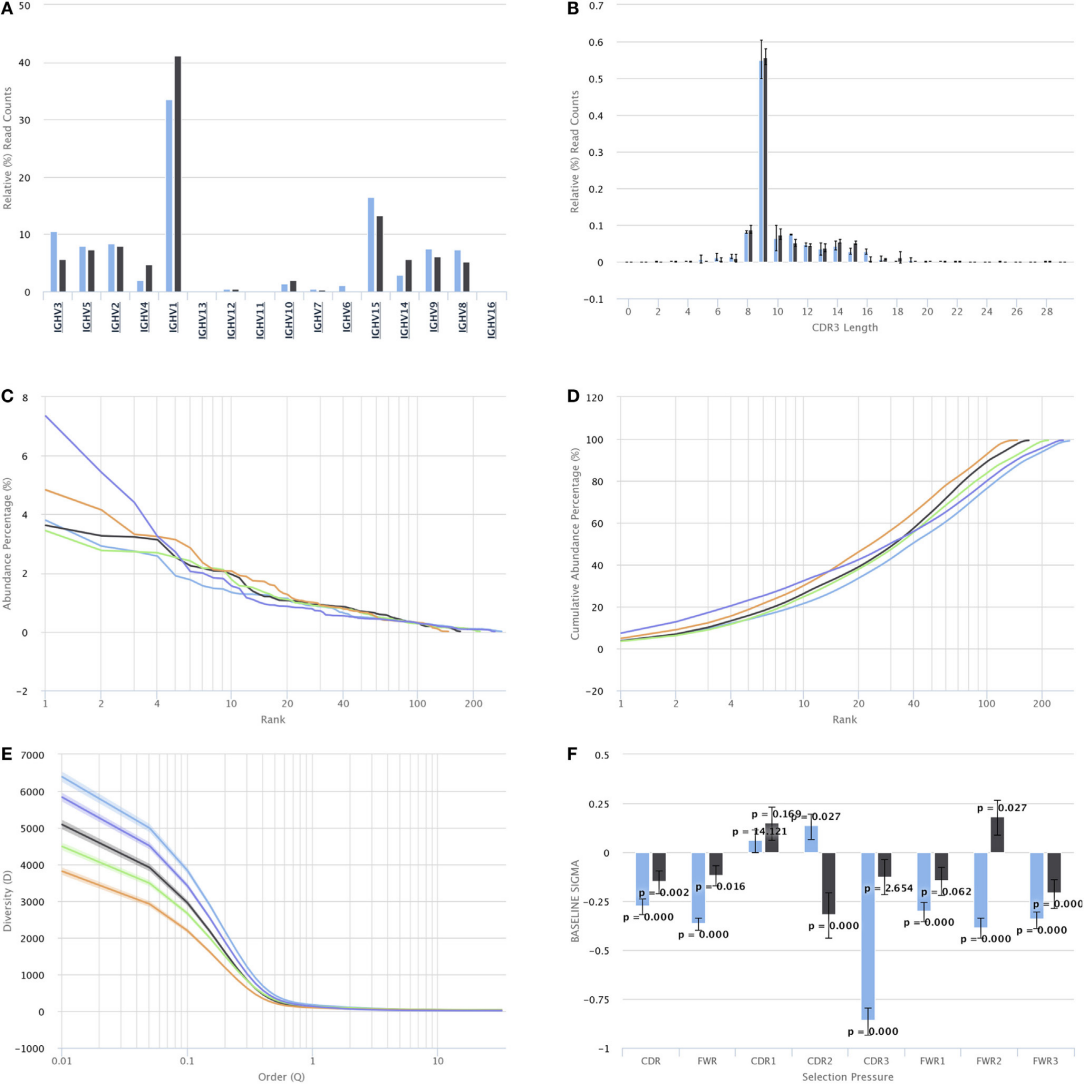
Repertoire characterization and comparison visualizations. For simplicity, the interface buttons for selecting which samples and sample groups to display are not shown. Full size versions of the figures are provided in Supplementary Material. **(A)** Gene segment usage histogram. The graph shows IGH V gene segments along the *X* axis and the percentage of reads assigned to each V gene segment along the *Y* axis. The percentage is currently being displayed for two samples, as indicated by the blue and black bars. **(B)** CDR3 length histogram. CDR3 length is shown on the *X* axis, and the percentage of reads with that CDR3 length is shown on the *Y* axis. The percentage is currently shown for two sample groups as indicated by the blue and black bars. When the data for sample groups, rather than samples, are displayed, the bar height represents the average percentage across all samples in the group, and the error bars indicate the SD. **(C)** Ranked clonal abundance percentage. Each colored line represents a sample with the clones ranked from highest abundance (rank 1) to lowest abundance along the *X* axis and the corresponding percentage of reads for each clone along the *Y* axis. **(D)** Cumulative clonal abundance. Each colored line represents a sample with clones ranked from highest abundance (rank 1) to lowest abundance along the *X* axis and the cumulative percentage along the *Y* axis. **(E)** Diversity profile. Each colored line represents a sample where clonal diversity along the *Y* axis is calculated across a sweep of the ordering parameter (*Q*) along the *X* axis. A point of *Q* = 1 corresponds to the Shannon entropy. **(F)** Quantification of selection pressure for CDR and framework regions. Region is shown on the *X* axis, and the value of the selection parameter is shown on the *Y* axis. Negative bar values indicate negative selection, and positive bar values indicate positive selection. The error bars show the 95% confidence interval of the selection parameter along with a *p*-value for significance. Two B cell samples are currently displayed as indicated by the blue and black bars.

For repertoire characterization and comparison, VDJServer provides visualizations for CDR3 length, gene segment usage, clonal composition, diversity, mutational distribution by position, and quantification of selection for framework and CDR regions. For gene segment usage, users can interactively drill down through the gene hierarchy and display counts at the locus (e.g., IGH or TRB), gene family, gene segment, and allele levels. Moreover, gene segment usage can display absolute counts, which is useful for magnitude analysis within a repertoire, and relative counts which is useful for comparison across repertoires. Clonal composition is visualized as a ranking of individual clones sorted by their relative abundance, or alternatively as a cumulative abundance curve of those sorted clones. Analysis results are displayed on each chart by selecting specific input files, or preferably by selecting repertoire samples and groups based upon the study metadata. Individual repertoire samples can be displayed together with repertoire groups on the same chart, with group results shown as an average with error bars or as a set of identically colored data elements. Examples of these charts are shown in Figure 5.

### Findable, Accessible, Interoperable, and Reusable (FAIR) Principles and Reproducibility

Computational analysis is becoming increasingly complex with multiple tools and steps involved in a typical workflow, and the exploratory nature of research often entails running multiple workflows to test alternative hypotheses and perform comparative analysis. The FAIR principles places emphasis on enhancing the ability of machines to find and use data, as well as the processes, tools, and workflows that led to that data (65). VDJServer supports these principles through its implementation with digital object identifiers (DOIs), an open and free API, standard formats, such as TSV and JSON, and detailed provenance. A DOI is a code to permanently and stably identify digital objects along with a standard mechanism to retrieve metadata about the object as well as access the digital object itself. Such digital objects in VDJServer include projects, files, study metadata, and jobs, all of which have a uuid (universally unique identifier) assigned to them. The Agave API, in combination with VDJServer’s API, allows digital objects to be accessed directly with their uuid or to be queried based upon the object’s metadata. The API URL with the object’s uuid provides a permanent and stable identification. Access to private data requires authentication while public data does not.

Replication of scientific results is vitally important, yet the burden of specifying the exact details of a computational analysis can easily lead to mistakes or missing information. VDJServer eliminates this burden by automatically capturing provenance for all jobs in a machine-readable JSON description of the inputs, parameters, and outputs of all computational processes. This provenance provides a trail for data as it is produced and passed from one computational tool to the next in an analysis workflow. Like other digital objects, computational provenance is given a uuid for access and can be queried. Furthermore, VDJServer can utilize the computational provenance to produce a detailed description of the complete analysis workflow for submission to a journal or database. Users are no longer burdened with remembering or keeping careful notes about exactly which tool was run to generate a particular set of results as VDJServer keeps track for them.

### High-Performance Computing

VDJServer provides free access to HPC at the Texas Advanced Computing Center (TACC). VDJServer automatically parallelizes tool execution based on the size of the input data. Computational analysis that takes days on a user’s desktop might take only a few hours on VDJServer. Small jobs are run on dedicated VDJServer machines and begin execution immediately, while larger jobs are queued to run on one of the TACC HPC clusters. Job submission to the HPC clusters is scheduled with optimized run time so that smaller jobs execute quickly, even if the queue is busy. At the time of this writing, VDJServer has processed billions of sequences through its analysis pipelines using TACC’s HPC resources.

## Results and Discussion

Immune repertoire analysis is having a significant impact across various research and clinical areas. VDJServer’s combination of an easy-to-use web interface, full suite of tools, interactive visualization, and HPC integration, facilitates a rapid, exploratory analysis cycle for scientific discovery. In the following sections, we describe two scientific use cases of VDJServer and the discoveries it has enabled.

### Use Case: Multiple Sclerosis

We used VDJServer to analyze the IGH repertoires of patients with relapsing–remitting multiple sclerosis (RRMS) in search of diagnostic immune biomarkers. The first study examined SHM patterns in the V gene segment (14), while the second study developed a statistical classifier based upon the CDR3 sequence (66). For both studies, B cells were obtained from the cerebrospinal fluid (CSF) of patients diagnosed with either RRMS or other neurological disease (OND). The first study also included CD19+ CD27− naïve B cells from the peripheral blood of one healthy donor. 454 sequencing was performed upon 40 samples for the first study (13 RRMS, 26 OND, 10 replicates from the single healthy donor), and an additional 102 samples for the second study (60 RRMS, 42 OND). All CSF samples were collected by lumbar puncture in accordance with IRB-approved protocols at UT Southwestern Medical Center, the University of Massachusetts Memorial Medical Center (UMass), Johns Hopkins University, or purchased from a commercial biorepository (PrecisionMed, Solana Beach, CA, USA).

The analysis workflow included VDJPipe for preprocessing, IgBlast for V(D)J assignment, and RepSum for rearrangement annotation. VDJPipe preprocessing trimmed the reads of primers and sample barcode sequences as well as filtered reads with average quality <35 or length shorter than 200 bp. Duplicate sequences were collapsed by VDJPipe with the resultant set of unique sequences input to IgBlast. RepSum annotation provided a secondary filtering step by eliminating sequences with frame-shifting insertions or deletions, out of frame junctions, stop codons present, truncated read lengths, less than 85% homology to germline sequences, missing CDR3s, or missing read coverage between Chothia-numbered codons 31 and 92. Also, unique sequence reads with fewer than two copies in the raw sequence data were discarded, as containing possible sequencing errors. Because sufficient B cells are not always obtained from CSF, samples were required to have at least 10 unique reads, otherwise they were excluded from further analysis. The filtering was performed with basic logical operations on the annotation fields produced by RepSum, thus providing a high-quality set of sequences for mutational analysis. VDJServer reports silent and replacement mutations at the nucleotide and codon level as part of the rearrangement annotation with replacement mutations indicating an amino acid substitution.

A second study applied machine learning to the CDR3 sequences within each sample repertoire to identify a biochemical motif present in the CDR3 sequences of RRMS repertoires but not OND repertoires (66). The analysis workflow was as described earlier, except pRESTO was used for preprocessing, and length filtering removed reads shorter than 300 bp. An additional step was performed to remove all reads shared between two or more samples. The remaining CDR3 sequences were used as input to the statistical classifier algorithm described in Ref. (66).

MS is an autoimmune disease that is notoriously difficult to diagnose, but early detection is needed because prompt intervention can significantly slow the progression of the disease. The VDJServer analysis was used for the development and refinement of a more accurate diagnostic test (MSPrecise) for RRMS based on the replacement mutation frequency for five V gene segment codons (14), and for the discovery of a biochemical motif present in the CDR3 sequences of RRMS repertoires but not OND repertoires (66).

### Use Case: Chimeric Antigen Receptor (CAR) T-Cells

Acute myeloid leukemia (AML) is the most common acute leukemia in adults, presenting greater than 20,000 new cases per year in the US and representing 80% of acute leukemias. The field of anti-cancer T-cell therapy has had major advances in recent years with the development of the CAR. A recent study examining AML has shown that a TIM3 monoclonal antibody blocks AML engraftment and eliminates leukemic stem cells (67). Those results, as well as others, led Davila and colleagues to hypothesize that anti-TIM3 CAR T-cells could target and kill AML. To test this hypothesis, mice were immunized with either Chinese hamster ovary (CHO) cells or CHO cells expressing TIM3. Immune repertoire sequencing was performed on splenocytes from both groups of mice, and repertoire comparison between the two groups was used to identify immunoglobulin (Ig) heavy and light chain genes that together form a receptor with TIM3 specificity. These genes were then used to develop novel, anti-TIM3 CARs. Animal studies were performed in accordance with the principles of the Basel Declaration and following protocols reviewed and approved by the Institutional Animal Care and Use Committee at the University of South Florida.

The Illumina sequencing platform was utilized to provide a total of over 18 million raw, paired-end sequence reads for Ig heavy and light chains from two control mice and three TIM3-treated mice. The analysis workflow included VDJPipe for preprocessing, IgBlast for V(D)J gene segment assignment, RepSum for rearrangement annotation, and RepCalc for repertoire comparison. preprocessing with VDJPipe merged the paired-end reads, filtered reads with average quality <25 or length shorter than 200 bp, and collapsed duplicate sequences. The resulting 12 million unique sequences for the five samples were given V(D)J gene segment assignments by IgBlast and annotated with RepSum. The analysis design entailed identifying rearranged Ig genes that were highly abundant in the TIM3-treated mice yet not present or very low in abundance in the control mice. We defined TIM3 and control sample groups using VDJServer’s study metadata entries, and RepCalc performed the repertoire comparison between the two sample groups. RepCalc performed both intragroup and intergroup comparison for V-J combinations, and V–J–CDR3 combinations, where the CDR3 was compared at both the amino acid and nucleotide sequence levels. RepCalc identifies shared and unique combinations and calculates abundance amounts for each sample and sample group. Sharing levels are provided that indicate how many samples within a sample group share a specific combination along with their abundance, and those sharing levels are also compared between sample groups. For example, a particular V–J combination might be present in one of three (intragroup unique), two of three (intragroup shared), or all three (intragroup shared) TIM3-treated mice, and one of two (intragroup unique) or all two (intragroup shared) control mice. While a combination that is present in at least one sample within a sample group but not present in any samples of the other sample group is intergroup unique, a combination that is present in at least one sample in both sample groups is intergroup shared. By analyzing the intragroup and intergroup comparisons along with their associated abundances, the Davila group could confidently identify rearranged Ig genes that were likely to respond to TIM3.

The use of immune repertoire sequencing with VDJServer analysis, in particular the detailed sharing levels produced by RepCalc, provides a rapid, economical system for the development of novel CARs that eliminated the need for hybridoma production and screening.

## Conclusion

VDJServer is a web-accessible cloud platform that provides access through a graphical user interface to a data management infrastructure, a collection of analysis tools covering all steps in an analysis, and an infrastructure for sharing data along with workflows, results, and computational provenance. VDJServer has been successfully used across a broad range of immune repertoire analysis projects, and is a free, publicly available, and open-source licensed resource.

VDJServer has been designed for long-term stability regardless of financial constraints that can often hamper research-oriented web resources. Specifically, VDJServer’s primary dependency is the cloud infrastructure provided by Agave (61), which is a core technology of the Texas Advanced Computer Center with a dedicated development team that is being continually enhanced and supports numerous other web resources besides VDJServer. The VDJServer-specific components are relatively small and easily fit on a small virtual machine, and the components have been containerized with Docker, which means the system can run on a user’s desktop if desired or run on a commercial cloud platform, such as Amazon Web Services or Microsoft Azure. Notably, Agave does not require use of TACC computing or storage resources. Because Agave abstracts the notion of execution systems (where jobs are run) and storage systems (where data are stored), users can define execution and storage systems that point to their own compute resources (or a commercial cloud platform) without loss of functionality, though the various analysis tools would need to be installed on the execution system. Nevertheless, in this scenario, the financial costs of the compute resources are shifted to the user.

VDJServer is continually advancing to meet the needs of the user community in the quickly developing field of immune repertoire analysis. Future goals include integrating new analysis tools and algorithms, providing additional interactive visualizations, enabling user queries across study metadata and rearrangement annotations, and conducting training and community outreach. With publication of the AIRR Minimal Information Standards (41), VDJServer currently ensures study metadata conforms to those standards. In the future, VDJServer will provide users the capability to submit their complete study, including project data, metadata, analysis, and results to a database in the International Nucleotide Sequence Database Collaboration [e.g., National Center for Biotechnology Information’s (NCBI) Sequence Read Archive and genetic sequence database (GenBank)] per the AIRR Minimal Standards recommendations. VDJServer will handle all of the technical details regarding data formatting requirements and standards compliance for submission, with accession numbers provided for journal publication. VDJServer will continue to evolve to remain consistent with AIRR standards as they are developed and released.

## Availability and Requirements

Project name: VDJServer
Project home page: https://vdjserver.org/
Source code repository: https://bitbucket.org/vdjserver
Docker images: https://hub.docker.com/r/vdjserver
Operating system(s): Platform independent
Programming language: JavaScript, Python
License: MIT, GNU GPL
Any restrictions to use by non-academics: no restrictions
No datasets were generated for this study.

## Ethics Statement

While the development of VDJServer did not involve the use of human or animal subjects, the two use cases presented to demonstrate VDJServer functionality did. The MS use case utilized human subjects. All samples were collected in accordance with IRB-approved protocols at UT Southwestern Medical Center, the University of Massachusetts Memorial Medical Center (UMass), Johns Hopkins University (JHU), or purchased from a commercial biorepository (PrecisionMed, Solana Beach, CA, USA). The protocols included an informed consent process. The CAR T-cells use case utilized animal subjects. All of the animal studies were performed in accordance with the principles of the Basel Declaration and following protocols reviewed and approved by the Institutional Animal Care and Use Committee at the University of South Florida.

## Author Contributions

SC, WS, ES, WR, IT, JF, ML, MK, SM, CJ, and JO designed, implemented, and tested the software code. AB and FR participated in testing through the web interface. MD and NM contributed driving use cases. NM, RS, and LC conceived the project, determined required user functionality, and provided feedback on the design. SC and LC wrote the manuscript. All the authors read and approved the final manuscript.

## Conflict of Interest Statement

The authors declare that the research was conducted in the absence of any commercial or financial relationships that could be construed as a potential conflict of interest. The reviewer NP declared a past co-authorship with the author to the handling Editor.

## Footnotes

^1^http://www.airr-community.org (Accessed: April 27, 2018).

^2^FastQC. Available from: http://www.bioinformatics.babraham.ac.uk/projects/fastqc (Accessed: April 27, 2018).

^3^ea-utils. Available from: https://github.com/ExpressionAnalysis/ea-utils (Accessed: April 27, 2018).

^4^AIRR Rearrangements File Format. Available from: http://docs.airr-community.org (Accessed: April 27, 2018).
