# Supplementary material for: VDJServer: A Cloud-Based Analysis Portal and Data Commons for Immune Repertoire Sequences and Rearrangements
Source: Front Immunol. 2018 May 8;9:976. doi: 10.3389/fimmu.2018.00976 (PMC5953328; doi:10.3389/fimmu.2018.00976)
Supplement: Supplementary file 1 [file Presentation_1.PDF]

Supplementary Material

VDJServer: A Cloud-Based Analysis Portal and Data Commons for Immune Repertoire Sequences and Rearrangements

Scott Christley, Walter Scarborough, Eddie Salinas, William H. Rounds, Inimary Toby, John Fonner, Mikhail K. Levin, Min Kim, Stephen A. Mock, Christopher Jordan, Jared Ostmeyer, Adam Buntzman, Florian Rubelt, Marco L. Davila, Nancy Monson, Richard H. Scheuermann, and Lindsay G. Cowell*

*** Correspondence:** Corresponding Author: lindsay.cowell@utsouthwestern.edu

1 Supplementary Data

1.1 Protocol for Basic Analysis Workflow

This protocol describes VDJServer Release V1.1. As new features are implemented, and new standards are developed by the AIRR Community, please visit https://vdjserver.org/docs for the latest documentation.

1.1.1 Create Account

All users need an account before they can upload files and run jobs. VDJServer uses a standard register and email verification process for new accounts.

1. Go to the main VDJServer web page at https://vdjserver.org/.
2. Click on the *Create Account* link to display the registration page.
3. Enter information in the form and submit. You need to provide a valid email address, username, and password.
4. You will receive an email verification at the specified email address. Click on the link to verify your account, after which you can immediately login to VDJServer.

A common error is not receiving the verification email. If it does not arrive in a few minutes, check any spam/junk folders as your email system might have put it there. If you cannot find the email, then send us feedback via the *Send Us Feedback* link on the main webpage or send an email to vdjserver@utsouthwestern.edu, and we can manually verify your account.

1.1.2 Create Project

Upon login, VDJServer will display the Project Management screen. As a new user, you have no projects, so the first step is to create a project. Each project is a logical container for files, jobs, analysis results, and visualizations, and any number of projects may be created. In the upper left corner of the window is an + *Add Project* link. Click on that link to display the add project screen, enter a project name, and submit to create a new project.

1.1.3 Upload Files

A new project has no files, so the *Upload and Browse Project Data* screen shows an analysis workflow overview figure by default. Near the top of the window is an *Upload* button that provides options to upload from your computer, upload from Dropbox, or upload from a URL (http/ftp), as shown in Supplementary Figure 12.

A. Uploading from your computer will provide a panel with the list of files to be uploaded, which will initially be empty. Click the *Add File(s)* button to open a File Browser window that allows you to pick the files on your computer, which will then be added to the list. You can click *Add File(s)* as many times as desired to select more files. You can also set some file attributes such as the *File Type* and *Read Direction* for the files, which can be more convenient than setting those attributes later. Once you've selected all the files to be uploaded, click the *Start* button to start uploading. The screen will present a progress bar during upload.
B. Uploading from Dropbox will present a Dropbox login screen, after which it will display your Dropbox files. Select the files to upload and click the *Choose* button, those files will begin uploading immediately.
C. Uploading from a URL will present a set of text fields where you can enter in the URL for the file(s). Up to ten URLs can be provided. Currently, VDJServer does not support URLs that require login.

Note that closing your browser window, browsing to another URL, turning off your computer, etc., will interrupt the uploading process. The speed of upload depends greatly upon the Internet connection of your computer and connection speed to VDJServer. If you have many large files to upload, it may be useful to upload files in small batches. Files can be compressed as zip, gzip, or bzip2 for faster upload; make sure the file has the appropriate extension (zip, gz, bz2) so VDJServer knows what compression was used.

1.1.4 Set File Attributes

Many VDJServer functions rely upon file attributes to determine appropriate inputs for analysis jobs and will also pass this information to tools so that they can properly process the data in the files. The most important attribute is the *File Type* as shown in Supplementary Figure 13. Current types include Read-Level Data, Barcode Sequences, Primer Sequences, Quality Scores, TAB-Separated Values, and VDJML. Read-Level Data is the primary type for immune repertoire sequencing data in FASTQ or FASTA format. Barcode Sequences and Primer Sequences are provided in FASTA format. The Quality Scores type is for immune repertoire sequencing data where the sequence reads and quality scores are provided in separate files (which is uncommon) with quality scores in QUAL format. TAB-Separated Values are used for Study Metadata spreadsheets, and VDJML is a sequence alignment format.

Set the appropriate *File Type* for each file. For sequencing data, also set the *Read Direction* attribute to indicate Forward or Reverse orientation. Forward is the appropriate orientation for reads sequenced from the V gene segment end of the template. Reverse is the appropriate orientation for reads sequenced from the J gene segment or constant region end of the template.

1.1.5 Link Sequencing Data Files

Paired-end read sequencing data is typically provided as two separate files; one file containing the forward reads and the other file containing the reverse reads. These two files need to be linked so that VDJServer and analysis tools know to process the two files together. This can be done on the *Link Paired-End Read Files* option for the project. Supplementary Figure 14 illustrates two pairs of read files that are linked together. Once files have been linked, they will be shown together as a pair on the project data screen as shown in Supplementary Figure 15.

Sequencing data that is provided as separate FASTA and QUAL files also need to be linked together. This can be done on the *Link FASTA/QUAL Files* option for the project. On that screen, select the appropriate Quality Score file that corresponds to the FASTA file. Once files have been linked, they will be shown together as a pair on the project data screen.

1.1.6 Run a VDJPipe Pre-Processing Job

With the appropriate file attributes defined and associated files linked together, it is now possible to run a pre-processing job. Only read data that contains quality scores is appropriate for pre-processing, so if your sequence data is all in FASTA format then proceed directly to the V(D)J Assignment step below. VDJServer currently provides pRESTO and VDJPipe as pre-processing tools. Both tools have similar functionality, though pRESTO supports Universal Molecular Identifiers (UMI), while VDJPipe runs significantly faster on large data sets. As pre-processing is a complicated process, we will not cover all of the possibilities; please review the documentation for pRESTO and VDJPipe for more details about specific functionality. We will demonstrate using VDJPipe.

1. From the project data screen, select the files that you want to provide as input to the pre-processing tool. Selection is done by clicking the small checkbox to the left of the file name.
2. Near the top of the screen is the *Run Job* button. Clicking on it will display a list of analysis tools that can be run. Select VDJPipe. This will display the initial job submission screen as shown in Supplementary Figure 16.
3. Enter in a job name or leave the default name, verify that the files you selected are shown, and select the appropriate workflow to be run. VDJPipe has many functions, so there are workflows that just perform single functions, and there is a complete workflow to run everything. Generally, the single function workflows are used to test different pre-processing parameters before running the complete workflow.
4. After selecting a workflow, the job submission screen will be updated to show the individual pre-processing modules that will be performed. Some modules have parameters, which have default values that you can change, while other modules don't have parameters and only describe what operation will be performed.
5. If any modules display a red warning box, it is important to either delete that module from the workflow by clicking the red X button, or to cancel the job submission and fix the error. If this is not done, then the job will attempt to run, but it will fail as it tries to perform that module. Supplementary Figure 17 shows an example of a red warning box for the Barcodes module. If you expect that barcode or primer pre-processing is to be performed but a red warning box is shown, that typically means you do not have the

Barcode or Primer file type properly set on the file. Cancel the job submission, set the file attributes, and re-run the job.

6. Once you've resolved any warnings and set appropriate parameters values, click the *Launch Job* button to submit the job to VDJServer.

1.1.7 Review Statistics with Pre-Processing Visualizations

The status of submitted jobs is shown on the *View Analyses and Results* screen for a project as demonstrated in Supplementary Figure 18. When a job has completed successfully, the *In Progress* label will change to the *View Output* button, and clicking it will display the output results from the job. For pre-processing jobs, the View Output screen has three main sections: Job Output Files, Analysis Charts, and Log Files. Job Output Files provides a list of output files generated from the pre-processing job. Analysis Charts provides visualizations for pre- and post-filtering statistics, and Log Files are job error logs as well as workflow provenance metadata.

The Analysis Charts are divided into a separate set of visualizations for each input file, so a pre-processing job with ten input files will have ten sets of analysis charts. There are five visualizations available, demonstrated in Supplementary Figures 1-5. You should review the pre- and post-filtering statistics on each chart to understand how pre-processing has affected your data. For example, if you imposed a minimum sequence length filter, then the Sequence Length Histogram chart will show you the distribution of read length from before and after pre-processing. Another example is setting a minimum quality filter, then both the Mean Quality Histogram and Quality Scores charts should be reviewed. If your pre-processing filters removed too many reads from your files, or alternatively have not removed enough, then you should run a new pre-processing job with looser or more stringent parameters. Among the Job Log files are summary logs that will give you information about the number of reads processed during each pre-processing step.

1.1.8 Make Job Output Files Available in Project Data Area

Once you are satisfied with the pre-processing results, you need to make the appropriate job output files available in the project data area so that they can be selected as input for additional analysis jobs. This is done from the *View Output* screen for the job. Next to each file in the Job Output Files section is a *Make Available in Project Data Area* button. Click this button to make that job output file show on the project data screen. That button acts as a toggle, it will change to *Remove from Project Data Area*, which when clicked will remove the job output file from the project data screen.

Some of the job output files represent intermediate steps in the pre-processing workflow, while one file is considered the final output. This can vary for different workflows. When running the complete workflow for VDJPipe, the final output file is called *Unique Post-Filter Sequences* and has the input filename shown in parentheses. Click on *Make Available in Project Data Area* for these files so that they can be passed as input to IgBlast in the next step. These job output files are grouped together on the project data screen as demonstrated in Supplementary Figure 19.

1.1.9 Perform V(D)J Assignment with IgBlast

The input files to IgBlast will either be job output files from a pre-processing job or uploaded FASTA files. Currently, VDJServer has separate germline databases for T-cells and B-cells and

for humans and mice so processing multiple cell types and/or organisms requires running multiple IgBlast jobs.

1. From the project data screen, select the files that you want to provide as input to IgBlast. Selection is done by clicking the small checkbox to the left of the file name.
2. Near the top of the screen is the *Run Job* button. Clicking on it will display a list of analysis tools that can be run. Select IgBlast. This will display the job submission screen as shown in Supplementary Figure 20.
3. Enter in a job name or leave the default name, verify that the files you selected are shown, select the appropriate organism, and select the appropriate sequence type.
4. Once you've set appropriate parameter values, click the *Launch Job* button to submit the job to VDJServer.

1.1.10 Define Study Metadata

In order to perform repertoire comparison, it is necessary to define the sample repertoires and sample groups for your study. This is done on the *Metadata Entry* screen for the project*. MiAIRR metadata consists of six components: study, subject, diagnosis, sample, cell processing, and nucleic acid processing. VDJServer adds a seventh component with sample groups. Each of these metadata components have their own data entry section on the *Metadata Entry* screen. Metadata can be manually entered on the screen but it is typically more efficient to prepare the metadata in a separate spreadsheet file, then import that spreadsheet into VDJServer. Here are the basic steps:

1. Go to the appropriate metadata section, click on the *Metadata Actions* button, and select *Export to File*. This will download a spreadsheet template file.
2. Open the spreadsheet template file in Excel. Use one row for each entry, and fill in values for each column. Refer to MiAIRR documentation regarding specific fields.
3. Save the completed spreadsheet file as a TSV (TAB-Separated Values) file. VDJServer cannot read native Excel spreadsheet files. Use *Save As…* to save the spreadsheet file in Excel, this will provide an option where you can specify the File Format as "Tab-delimited Text".
4. Upload the completed spreadsheet file into the *Project Data Area*. Make sure to set the File Type to TAB-Separated Values.
5. On the *Metadata Entry* screen, go to the appropriate metadata section, click on the *Metadata Actions* button, and select *Import from File*. This will show a panel where you can select the appropriate file as demonstrated in Supplementary Figure 21.
6. The panel will indicate when the importing has finished and re-display the *Metadata Entry* screen. You can still manually alter the metadata, if desired, or import additional files.

Sample groups are a specialized feature of VDJServer that allows sample repertoires to be grouped together for performing intra-group and inter-group comparisons. Supplementary Figure 22 shows four sample groups which have been defined. Sample groups are defined by using one or more grouping operations. These grouping operations include:

1. Grouping by the values of a study metadata field. The *Group By* option provides a popup list of all the possible fields. VDJServer will determine all of the values for that field

among the study metadata and create a separate group for each value. For example, if you have sample repertoires for two cell subsets, CD4 and CD8, then two sample groups will be created by selecting the sample metadata cell_subset field.
2. Grouping by a logical operation. The *Logical* option allows you to define a simple Boolean expression. Sample repertoires where that expression evaluates as true will be included in the sample group, while those for which the expression evaluates as false will be excluded. Pick the study metadata field from the popup list of available fields, pick the comparison operator, and provide a value. VDJServer will automatically handle strings versus numbers, depending upon how the MiAIRR field is defined.
3. Individual samples can be picked. The *Repertoires* option provides a popup list of sample repertoires. By default, when no sample repertoires are selected, then all sample repertoires are included in the sample group. Click on specific sample repertoires in the list to include them in the sample group.
4. The three grouping operations can be combined together. A sample repertoire needs to satisfy all applicable grouping operations to be included in the sample group. For example, in Supplementary Figure 22, a sample group is defined with *Group By* option for cell_subset and with a *Logical* expression (age = 27yr).

*Note: VDJServer currently defines study metadata according to the MiAIRR standard, which is quite extensive, and primarily directed towards manuscript submission. The AIRR Community is actively working on additional metadata standards that are more appropriate for analysis workflows. When VDJServer implements any of these new standards, existing metadata will automatically be converted for users.

1.1.11 Perform Repertoire Characterization and Comparison with RepCalc

RepCalc requires a variety of different output job files from IgBlast in order to perform its calculations. Instead of requiring the user to properly make all of the appropriate files available in the project data area, where mistakes can lead to subtle errors, RepCalc will access the files it needs directly from the IgBlast job output. This means, however, that all sample repertoires that you wish to analyze together with RepCalc must be processed within a single IgBlast job.

In the previous section, we described defining study metadata to perform repertoire comparison between sample repertoires and sample groups. However, RepCalc does not require study metadata. If none is provided, then RepCalc will perform calculations and comparisons on a file-by-file basis. RepCalc performs numerous calculations including gene segment usage, CDR3 analysis, clonal assignment and analysis, diversity calculations, and mutation analysis. By default, all of the calculations are performed, but any can be disabled or altered on the job submission screen.

1. No files need to be selected on the project data screen in order to run RepCalc.
2. Near the top of the screen is the *Run Job* button, clicking on it will display a list of analysis tools that can be run, select RepCalc. This will display the job submission screen as shown in Supplementary Figure 23.
3. Enter in a job name or leave the default name, verify that your study metadata is recognized, and select the IgBlast job with the sample repertoires to analyze. The remaining sections of the job submission screen contain settings for different analysis modules.

4. Once you've set appropriate parameters values, click the *Launch Job* button to submit the job to VDJServer.

1.1.12 Review Analysis Visualizations

The status of your RepCalc jobs is shown on the *View Analyses and Results* screen for a project. When the job has completed successfully, click on the *View Output* button to display the output results from the job. For RepCalc jobs, the View Output screen has three main sections: Job Output Files, Analysis Charts, and Log Files. Job Output Files provides a list of clonal assignment output files, Analysis Charts provides analysis visualizations, and Log Files are job error logs as well as workflow provenance metadata.

RepCalc produces a set of eight interactive analysis charts, as illustrated in Supplementary Figures 6-11. Gene segment usage has one chart showing absolute counts and another chart showing relative counts, while CDR3 length distribution has one chart for amino acid and another for nucleotide. If a particular analysis was de-selected on the job submission screen, then it's corresponding analysis chart will not be displayed. Each chart provides three pop-up lists for selecting files, sample repertoires, or sample groups to be displayed on the chart. Supplementary Figure 24 demonstrates picking two sample groups to be displayed together on the relative gene segment usage chart. Chart figures can be downloaded by clicking on the *Download Chart* button, which will generate a figure identical to the chart being displayed in the browser. Also, the raw data can be downloaded by clicking on the *Download Data* button. All of the raw data is provided in TSV format for easy import into Excel and other tools.

1.1.13 Downloading Data Files

Throughout the analysis workflow process, there are numerous opportunities to download data files associated with steps in the workflow. Such data files include files that were originally uploaded into a project, job output files, analysis result data that underlies the visualization charts, and job log files. We describe how to download each in the following:

A. Files that were originally uploaded into a project can be downloaded from the *Upload and Browse Project Data* screen. Each file name is a button, which when clicked will download the file.
B. Job output files can be downloaded by first going to the *View Analyses and Results* screen for a project and clicking on the *View Output* button to display the output results from the job. In the Job Output Files section, each file name is a button, which when clicked will download the file.
C. Analysis results data that underlie the visualization charts can be downloaded by first going to the *View Analyses and Results* screen for a project and clicking on the *View Output* button to display the output results from the job. In the Analysis Charts section, click on the *Download Data* button to download all of the analysis results data for that chart.
D. Job log files can be downloaded by first going to the *View Analyses and Results* screen for a project and clicking on the *View Output* button to display the output results from the job. In the Log Files section, each file name is a button, which when clicked will download the file.

E. If you want most or all of the job output files, it may be tedious to download each one-by-one. Alternatively, you can download all of the files together. Go to the *View Analyses and Results* screen for a project and click on the *View Output* button to display the output results from the job. In the Log Files section, there is a file called Archive of Output Files, click on this file to download a zip-compressed archive containing the job output files, analysis results files, and the log files for the job.

2 Supplementary Figures and Tables

2.1 Supplementary Figures

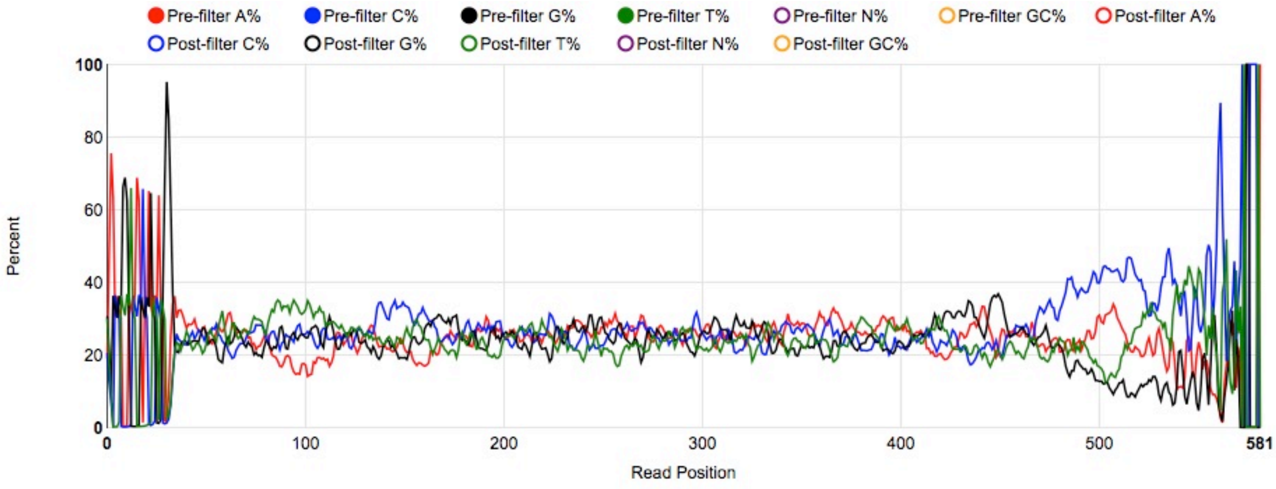

Supplementary Figure 1. Pre-processing visualization. Nucleotide composition for each read position. Each colored line represents the percentage of a nucleotide or ambiguous base call (N) at each read position. The legend functions as a toggle to set which lines are shown. The figure is currently showing the composition pre-filtering for A, C, G, and T nucleotides.

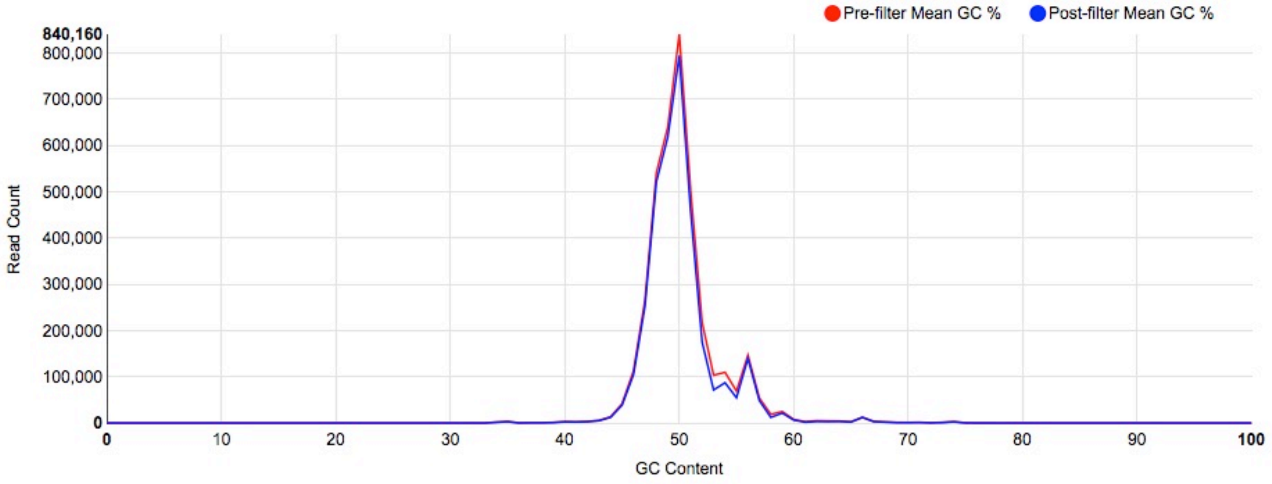

Supplementary Figure 2. Pre-processing visualization. GC content histogram. The graph shows the number of reads along the Y axis and GC percentage along the X axis. The red curve indicates the number for pre-filter reads, and the blue curve represents the number for post-filter reads.

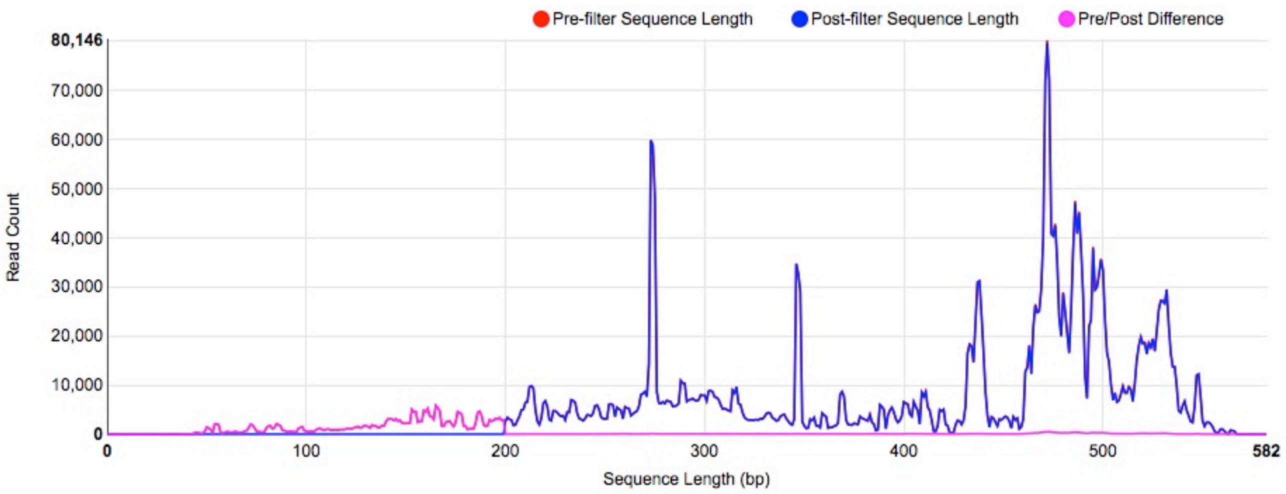

Supplementary Figure 3. Pre-processing visualization. Sequence length histogram. The graph shows the number of reads along the Y axis and sequence length along the X axis. The red curve shows the number of sequences of each length for pre-filter reads, the blue curve shows the number for post-filter reads, and the magenta curve shows the pre/post difference. The graph clearly shows that a length filter of 200 was used.

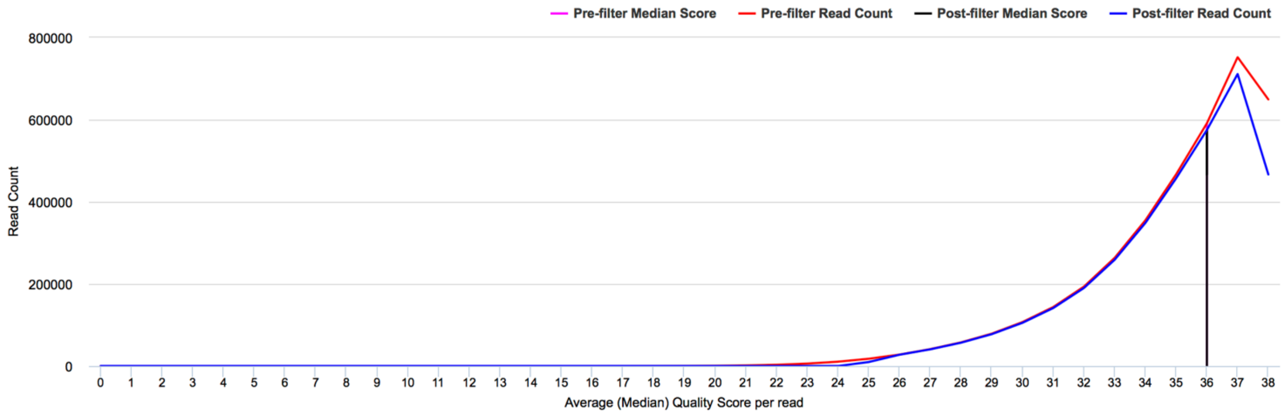

Supplementary Figure 4. Pre-processing visualization. Mean quality score histogram. The graph shows the number of reads along the Y axis and the average quality score along the X axis. The red curve shows the number of sequences for pre-filter reads, and the blue curve shows the number for post-filter reads. The magenta and black vertical lines show the median score (36) for the pre- and post-filter reads, respectively.

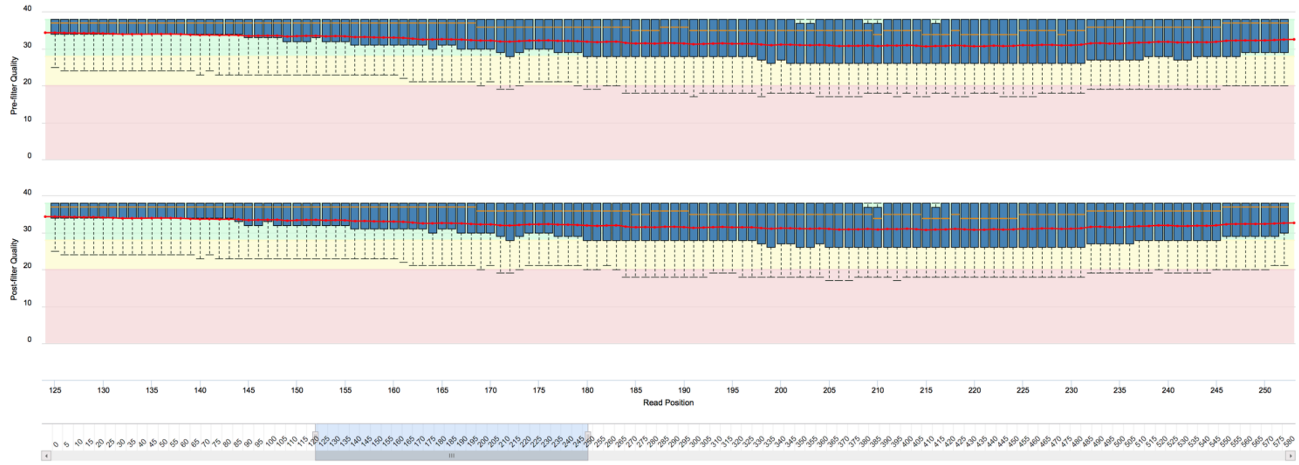

Supplementary Figure 5. Pre-processing visualization. Quality score distribution for each read position. Quality score is shown on the Y axis, and read position is shown on the X axis. At each position, the box and whiskers plot shows the median quality score, inter-quartile range, and the 10th and 90th quantiles. The X axis has zoom control, set to positions 125 – 250 in the current figure.

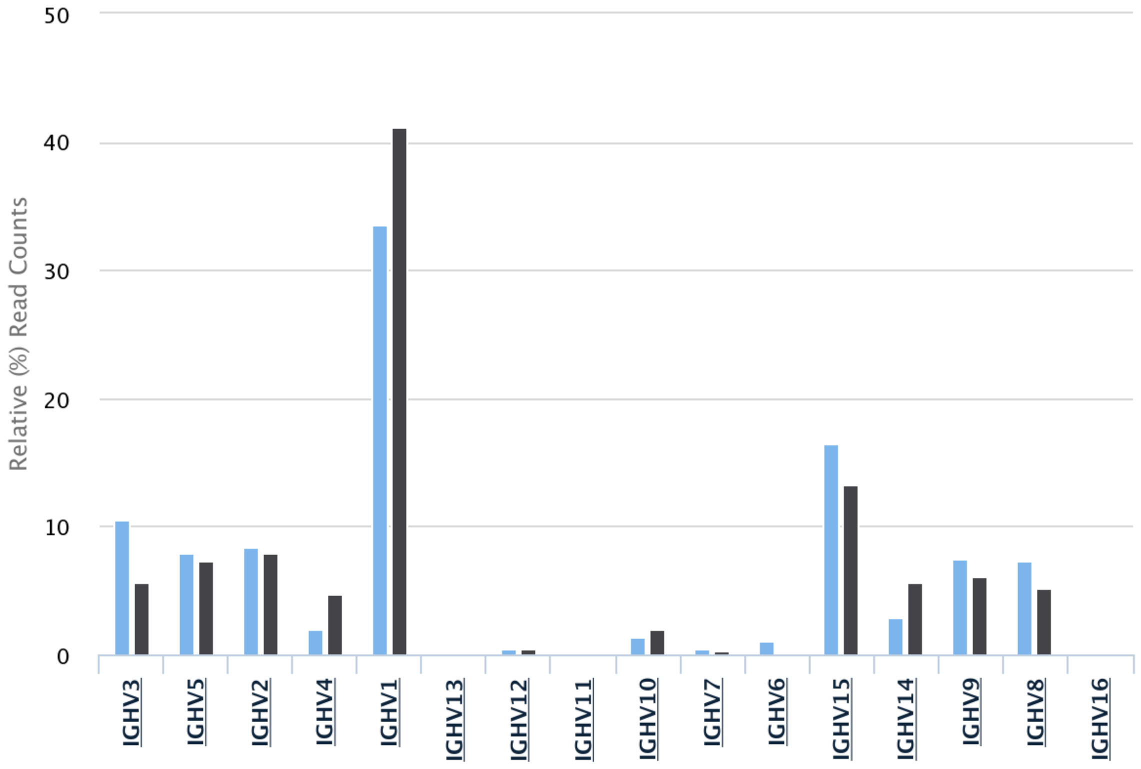

Supplementary Figure 6. Repertoire characterization and comparison visualization. Gene segment usage histogram. The graph shows IGH V gene segment type along the X axis and the percentage of reads assigned each V gene segment along the Y axis. The percentage is currently being displayed for two samples, as indicated by the blue and black bars.

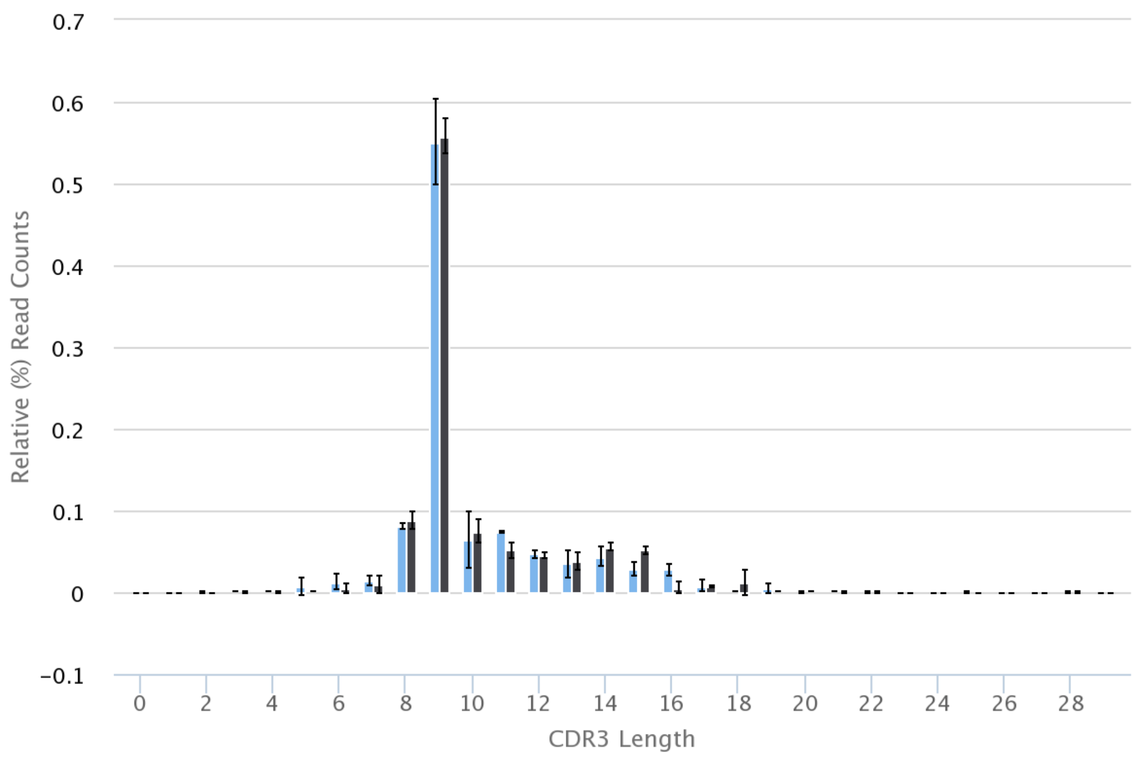

Supplementary Figure 7. Repertoire characterization and comparison visualization. CDR3 length histogram. CDR3 length is shown on the X axis, and the percentage of reads with each CDR3 length is shown on the Y axis. The percentage is currently shown for two sample groups as indicated by the blue and black bars. When the data for sample groups, rather than samples, is displayed, the bar height represents the average percentage across all samples in the group, and error bars indicate the standard deviation.

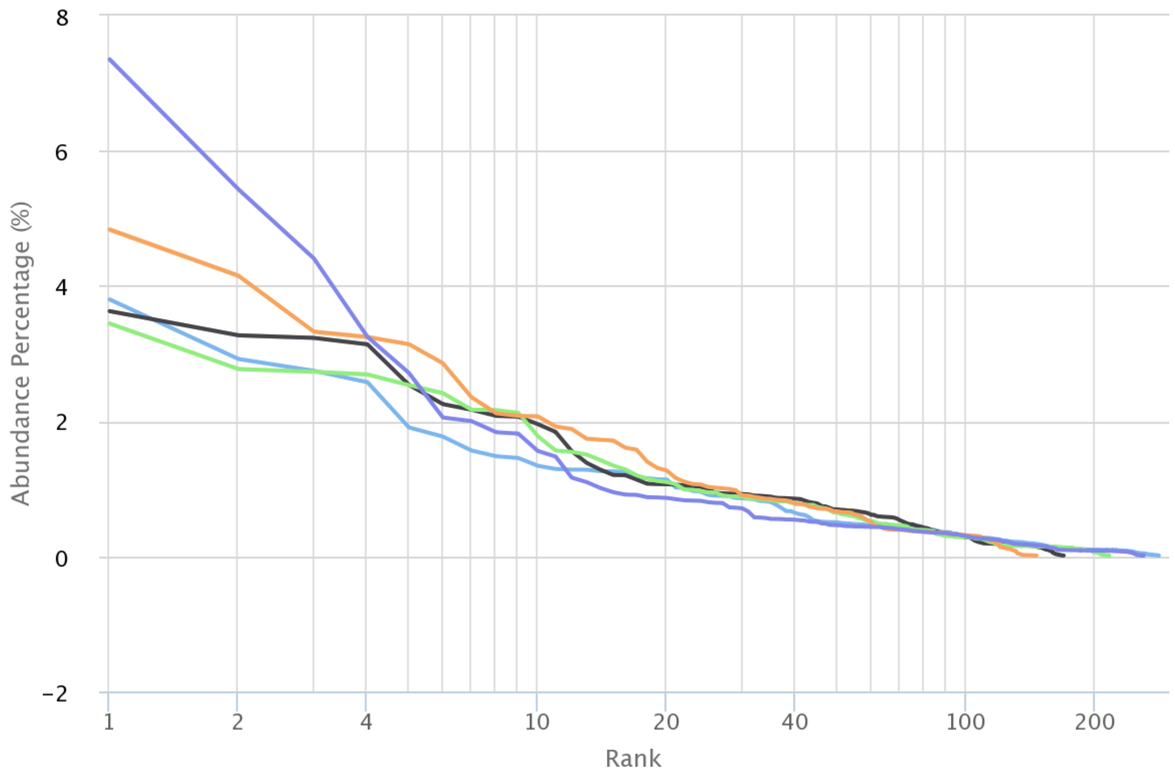

Supplementary Figure 8. Repertoire characterization and comparison visualization. Ranked clonal abundance percentage. Each colored line represents a sample with the clones ranked from highest abundance (rank 1) to lowest abundance along the X axis and the corresponding percentage of reads for each clone along the Y axis.

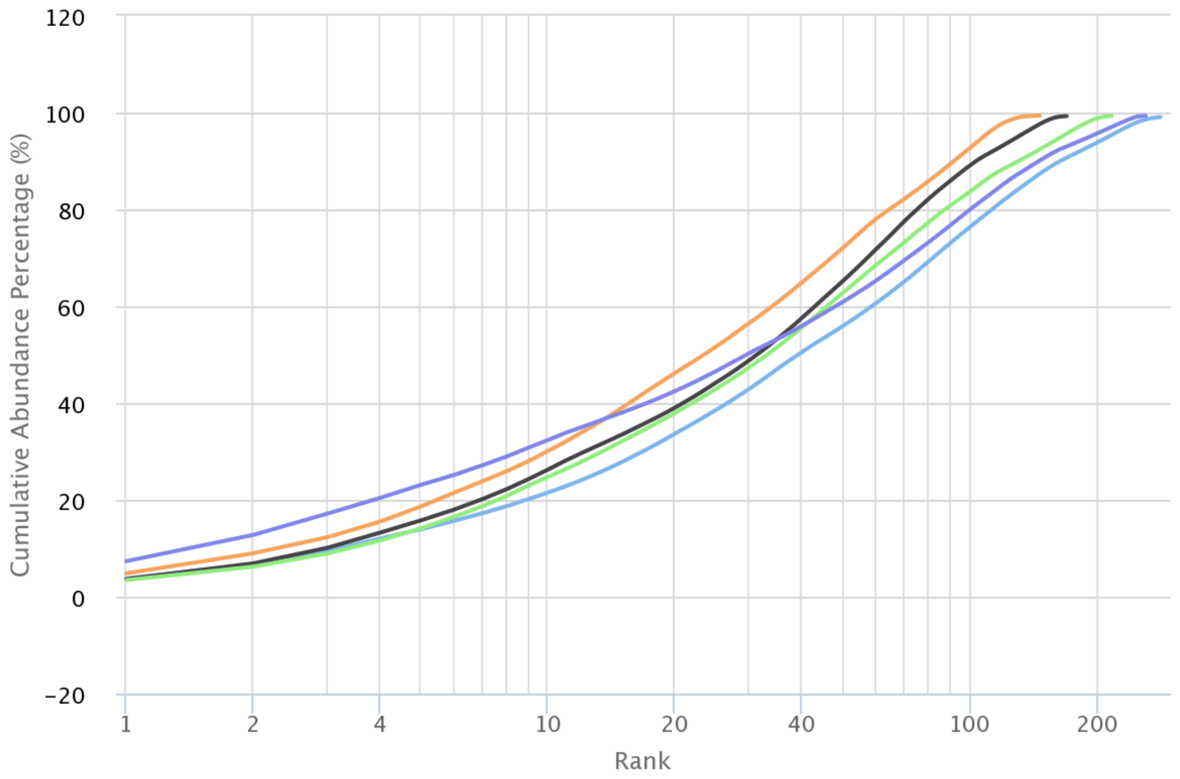

Supplementary Figure 9. Repertoire characterization and comparison visualization. Cumulative clonal abundance. Each colored line represents a sample with clones ranked from highest abundance (rank 1) to lowest abundance along the X axis and the cumulative percentage along the Y axis.

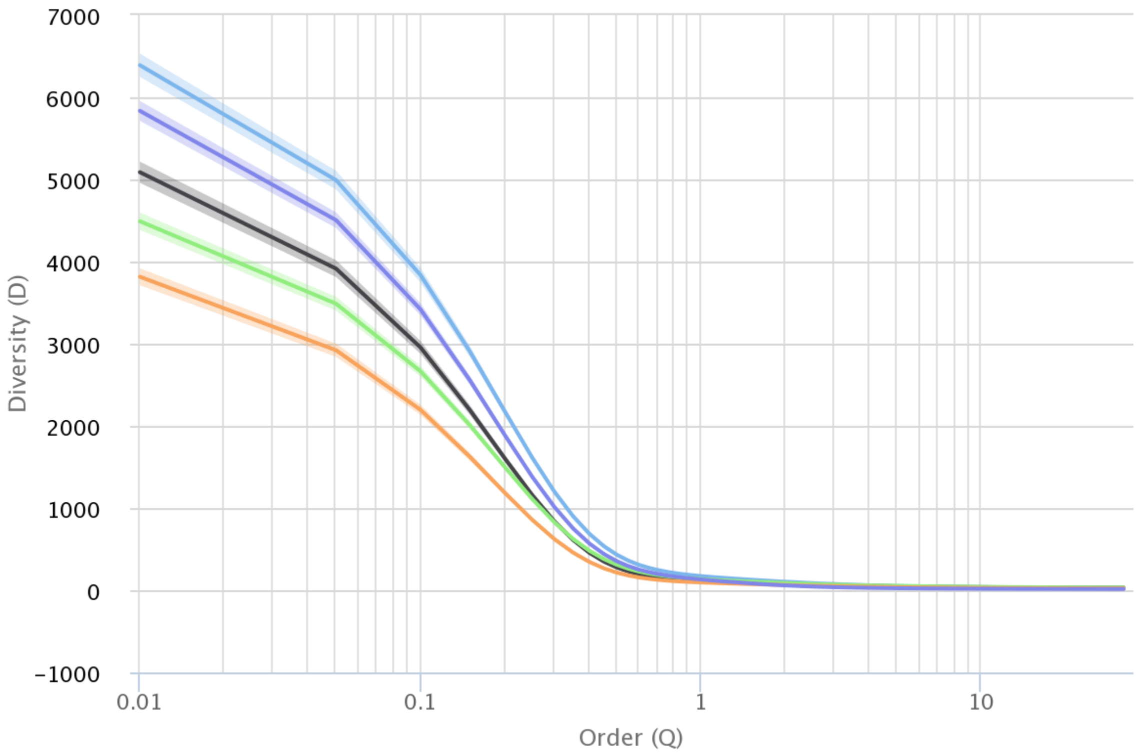

Supplementary Figure 10. Repertoire characterization and comparison visualization. Diversity profile. Each colored line represents a sample where clonal diversity along the Y axis is calculated across a sweep of the ordering parameter (Q) along the X axis. A value of Q=1 corresponds to the Shannon entropy.

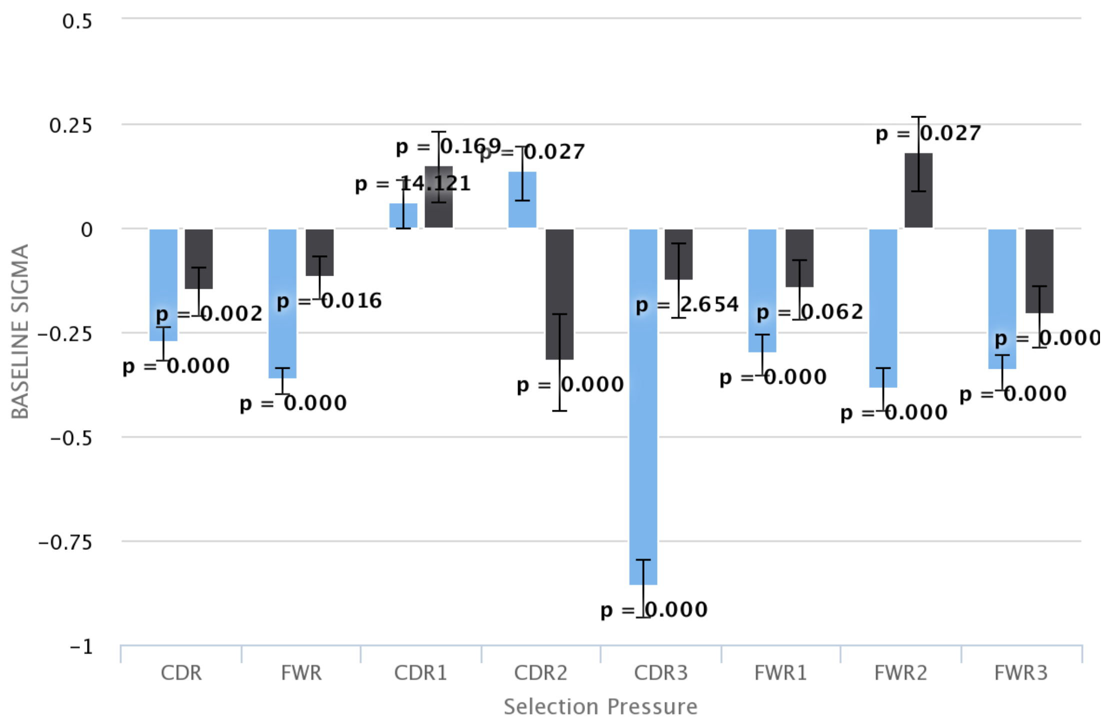

Supplementary Figure 11. Repertoire characterization and comparison visualization. Quantification of selection pressure for CDR and framework regions. Region is shown on the X axis, and the value of the selection parameter is shown on the Y axis. Negative bar values indicate negative selection, and positive bar values indicate positive selection. The error bars show the 95% confidence interval of the selection parameter along with a p-value for significance. Two B cell samples are currently displayed as indicated by the blue and black bars.

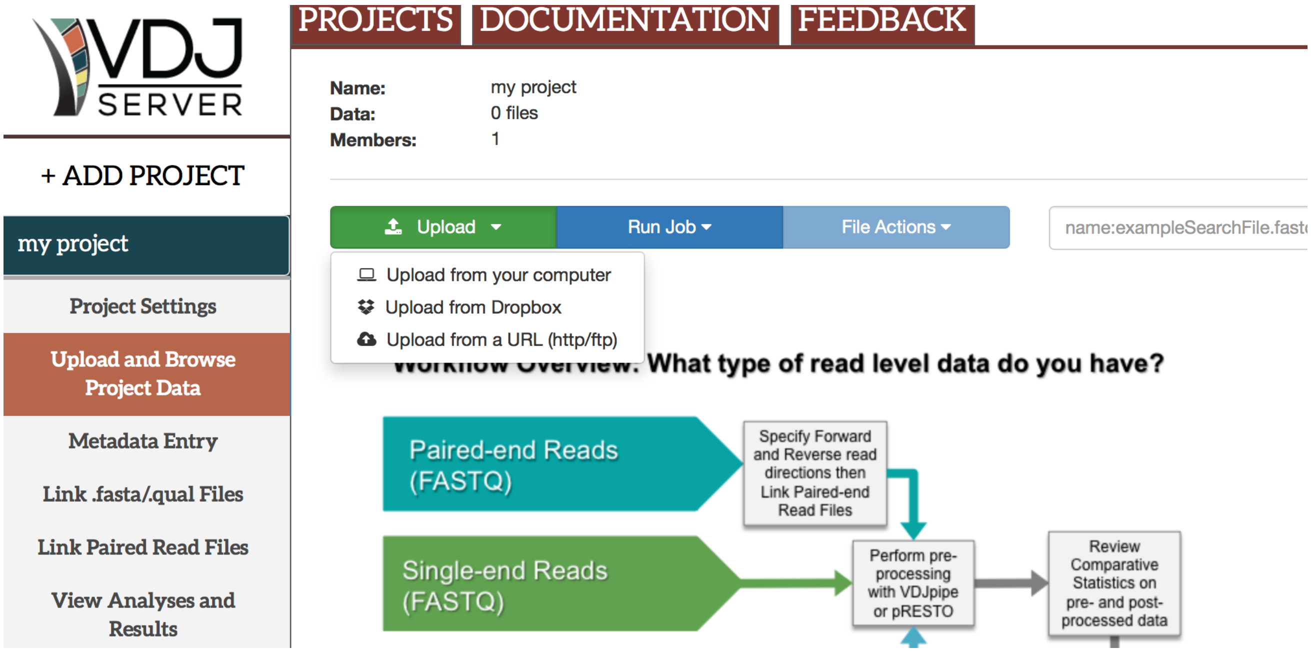

Supplementary Figure 12. Uploading files into a project. The green Upload button is available on the *Upload and Browse Project Data* screen for the project, which when clicked, reveals a clickable list of file locations from which to upload. Files can be uploaded from your computer, from Dropbox, or from a URL (http/ftp).

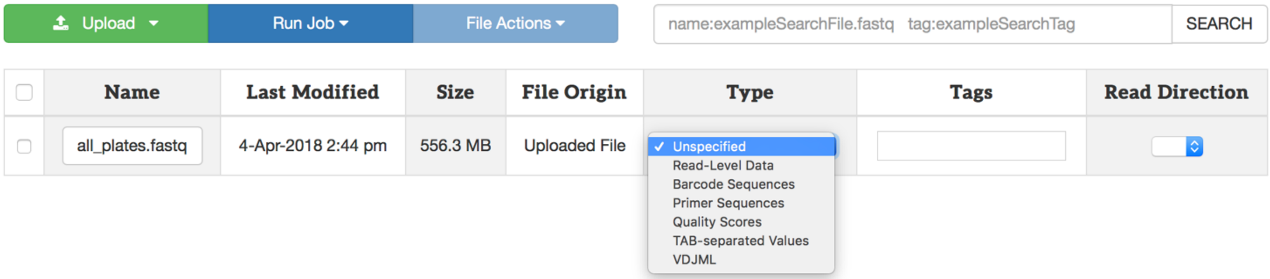

Supplementary Figure 13. Setting File Type attributes for files. VDJServer uses attribute information for displaying the appropriate files on various screens, and analysis tools might use this information to properly process the data in the file.

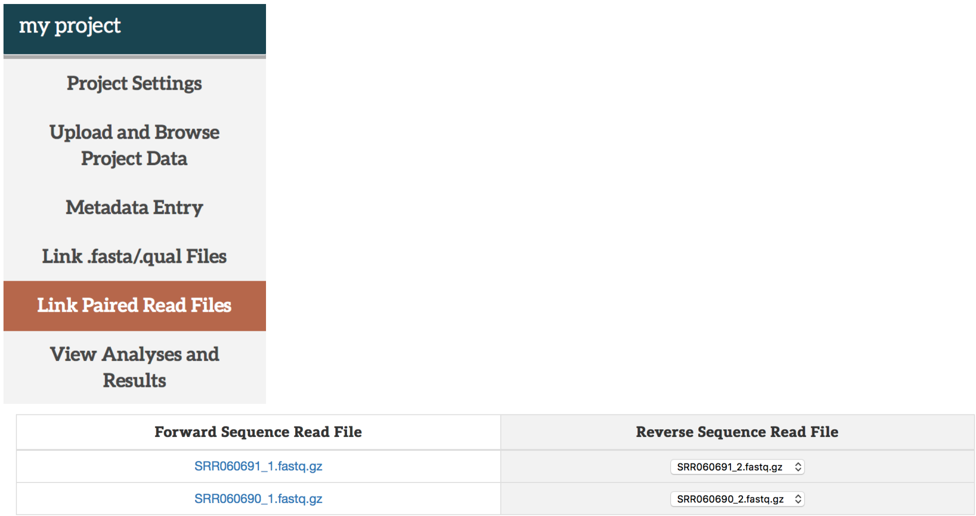

Supplementary Figure 14. Link Paired Read Files. The screen only shows files of type Read-Level Data with the appropriate Forward or Reverse read direction.

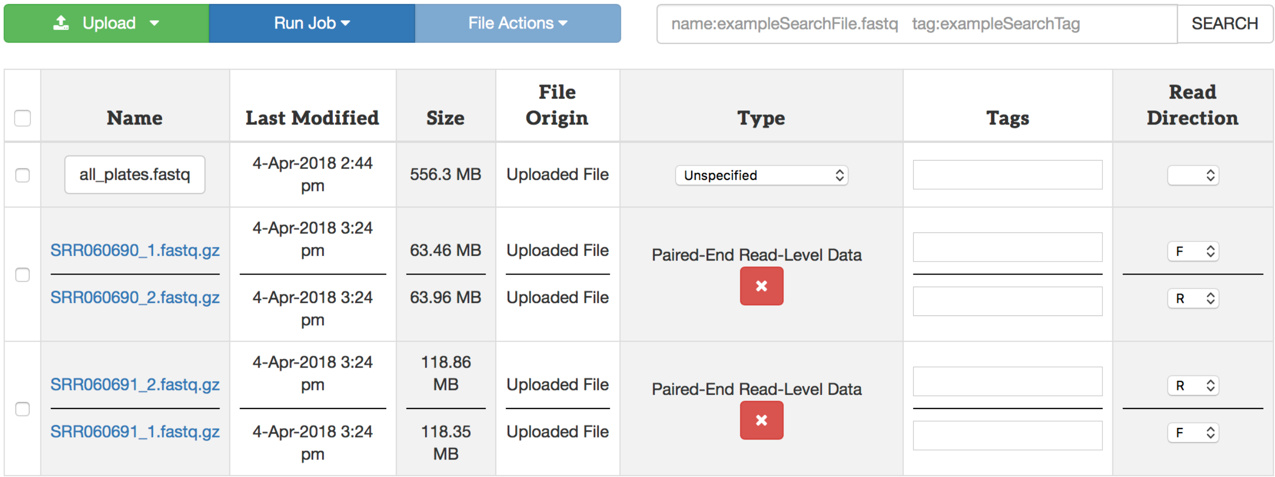

Supplementary Figure 15. Linked files are shown together on the *Upload and Browse Project Data* screen.

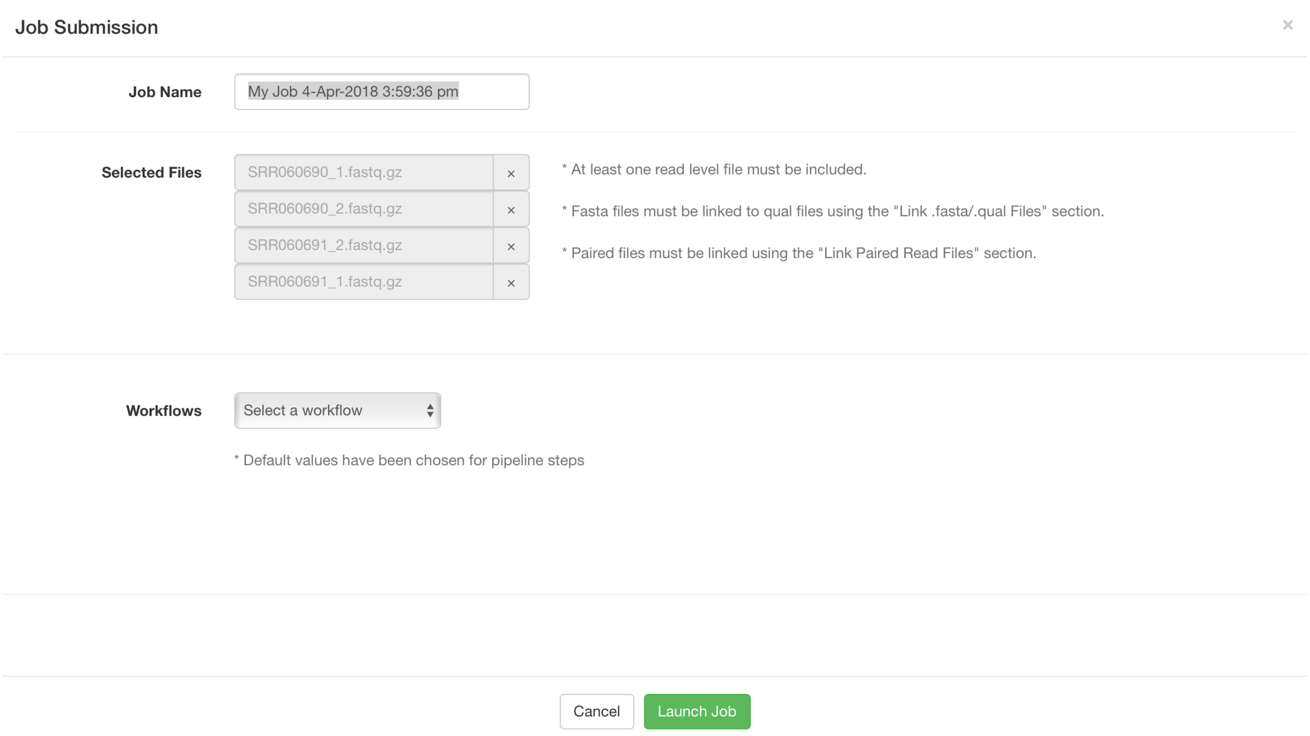

Supplementary Figure 16. Initial job submission screen for VDJPipe. Once a workflow is selected, then more detailed job submission parameters will be displayed.

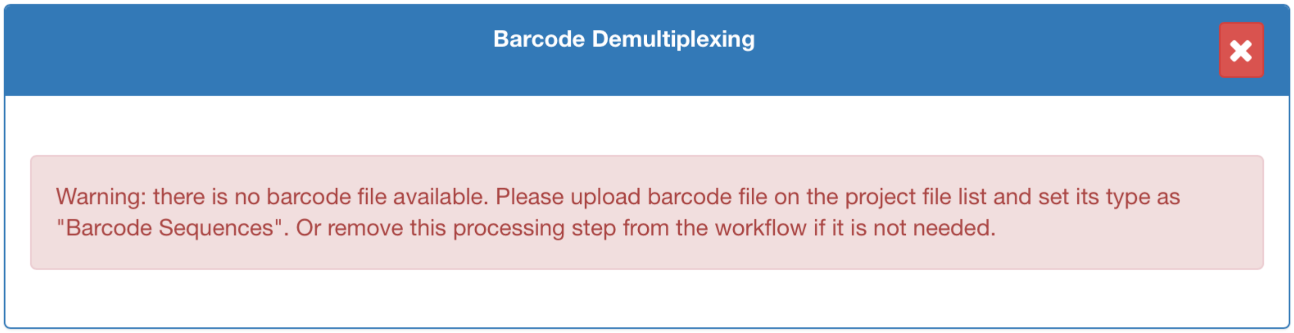

Supplementary Figure 17. Red warning box indicating that VDJServer cannot find any file of type Barcode Sequences, which is required input to the Barcode Demultiplexing module of VDJPipe. You need to either click the red X in the upper right corner to delete the module from the workflow, or cancel the job submission and set the file type on the appropriate files in project data.

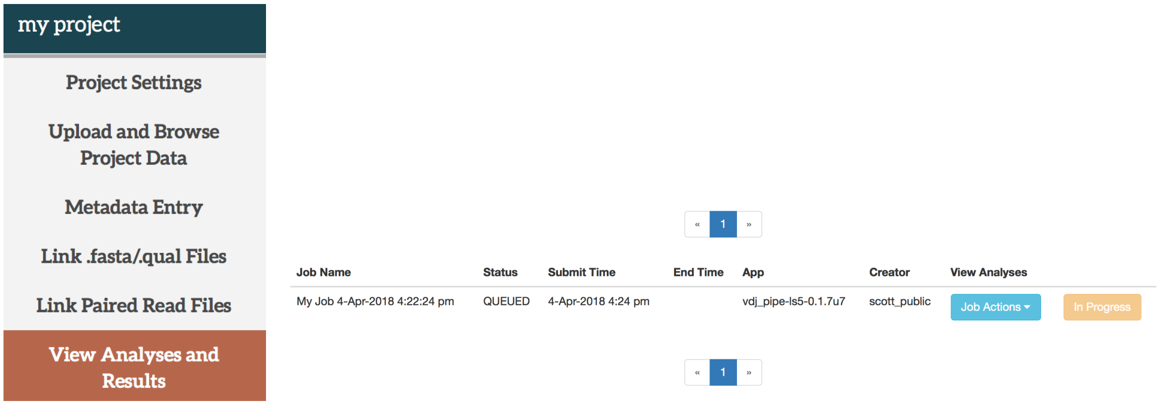

Supplementary Figure 18. Status of submitted jobs. When a job has completed successfully, the *In Progress* button will change to *View Output*, and clicking it will display the output results from the job.

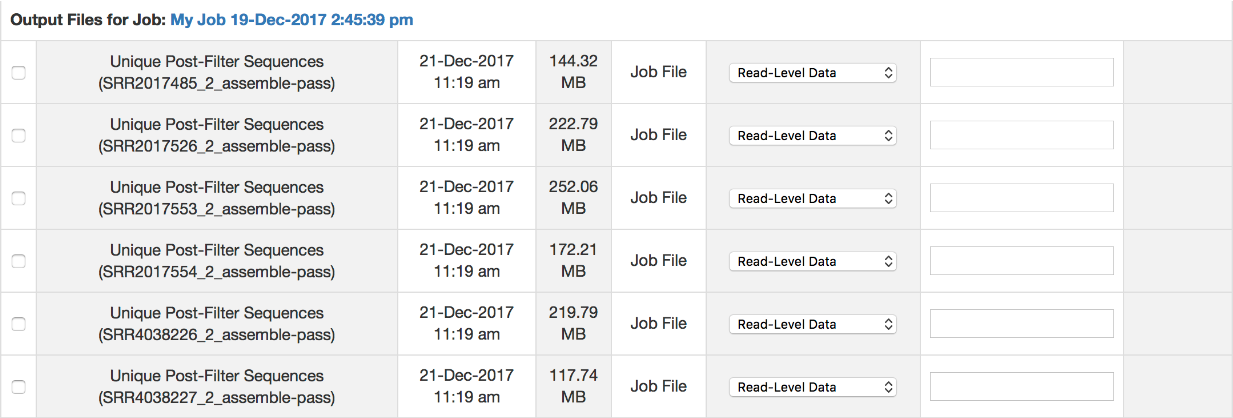

Supplementary Figure 19. Job output files that have been made available in the project data area so that they can be selected as input for other analysis jobs.

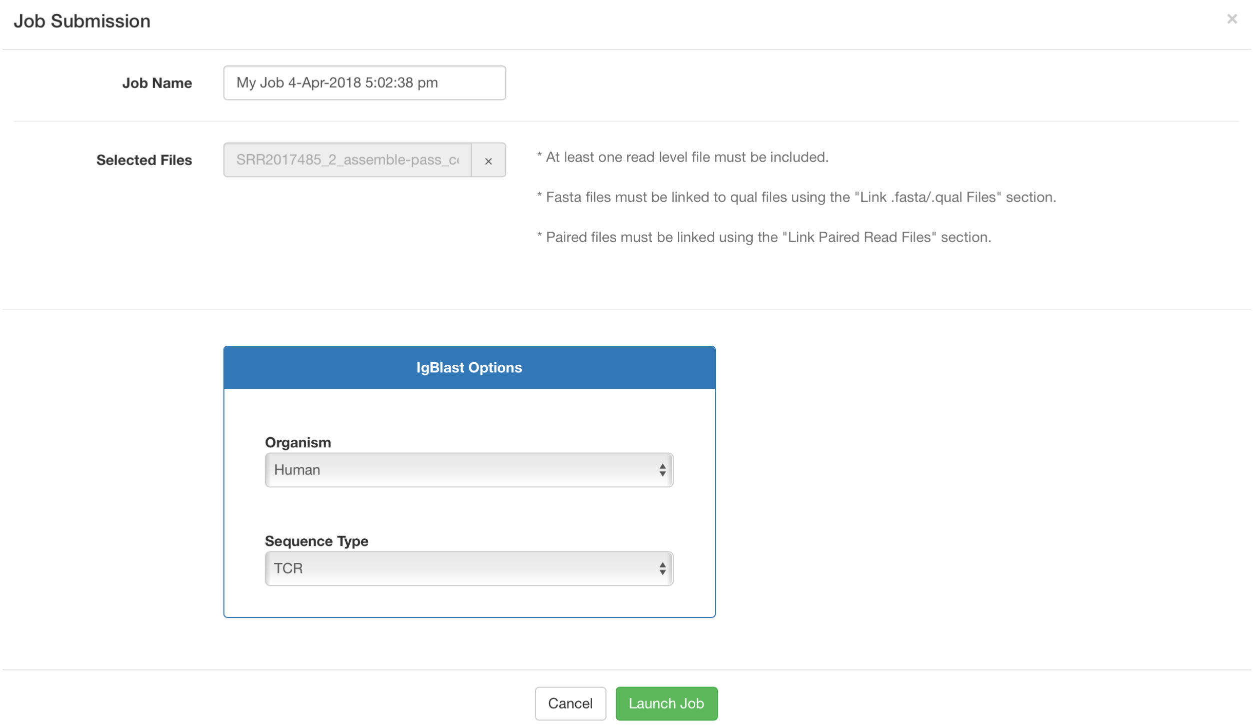

Supplementary Figure 20. IgBlast job submission screen. Shown options are set to perform V(D)J assignment for human T-cell receptor sequences.

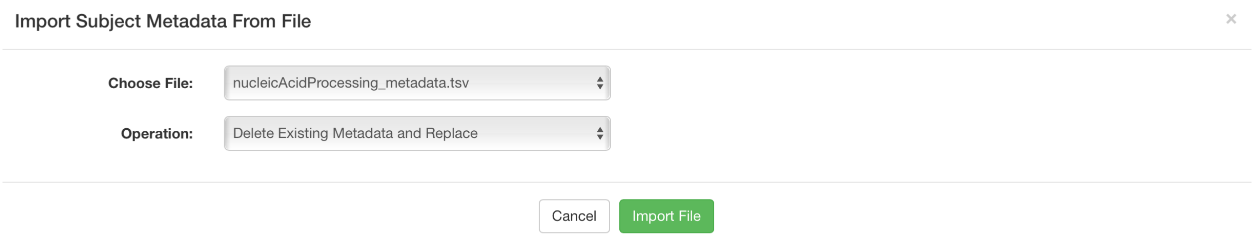

Supplementary Figure 21. Import Metadata from File window. Choose the appropriate TAB-Separated Values file from the project data area and indicate whether the metadata should replace or be appended to the existing metadata.

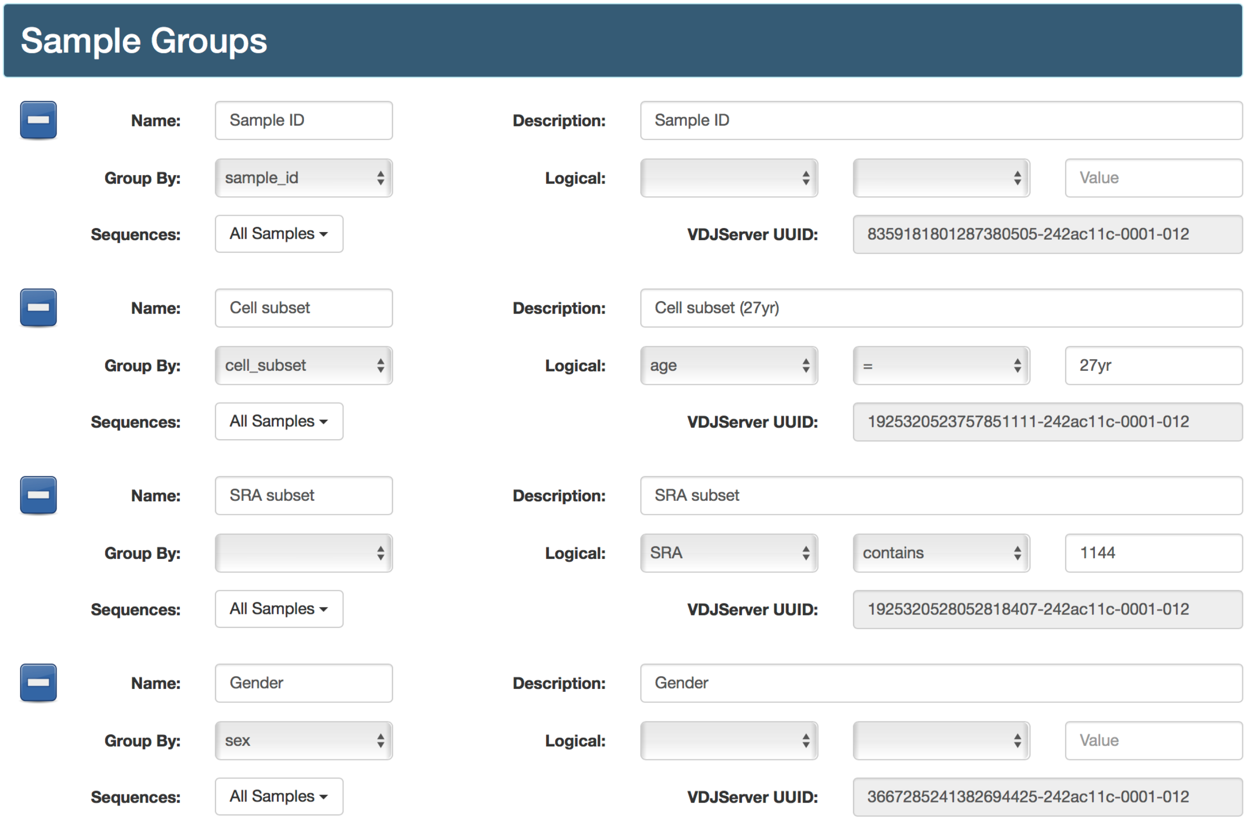

Supplementary Figure 22. Sample Groups. Four sample groups are defined using a variety of grouping operations. The first grouping will form sample groups based on Sample ID. Since Sample IDs should be unique to each sample, each sample group will have only one sample in it. The second grouping will form sample groups based on the cell subset of the sample, including only samples from donors of 27 years of age. The third grouping will form groups based on the samples that contain "1144" as a substring in the SRA accession number. The fourth grouping will form sample groups based on the biological sex of the sample donor (referred to as gender in the sample metadata for this data set). Samples derived from males will form one group, and samples derived from females will form a second group. If additional values are included (such as unknown), those will also form groups.

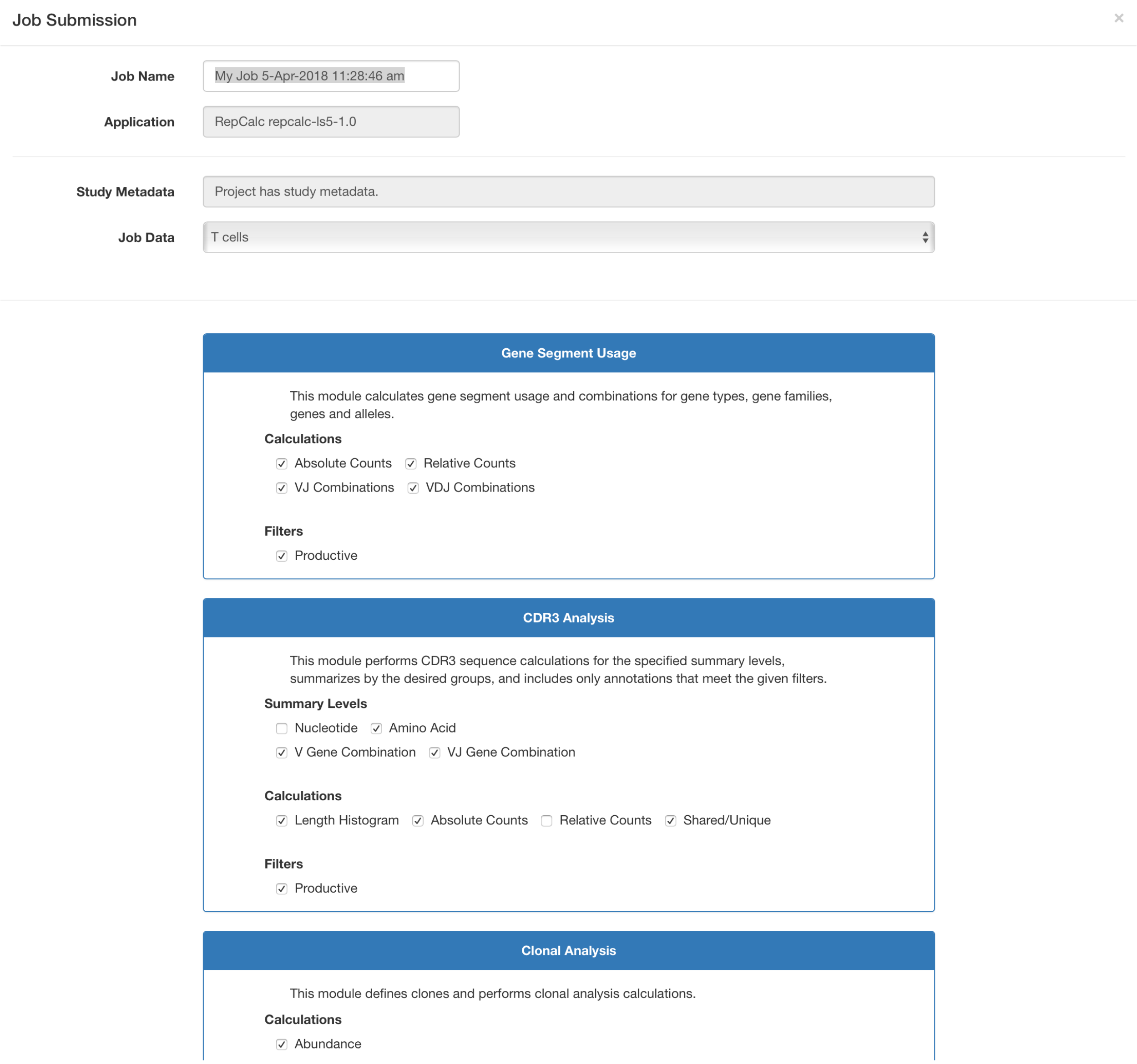

Supplementary Figure 23. RepCalc job submission screen. This project has study metadata defined as indicated, and the IgBlast job “T cells” is selected. Each analysis module has a section with settings for calculations to be performed.

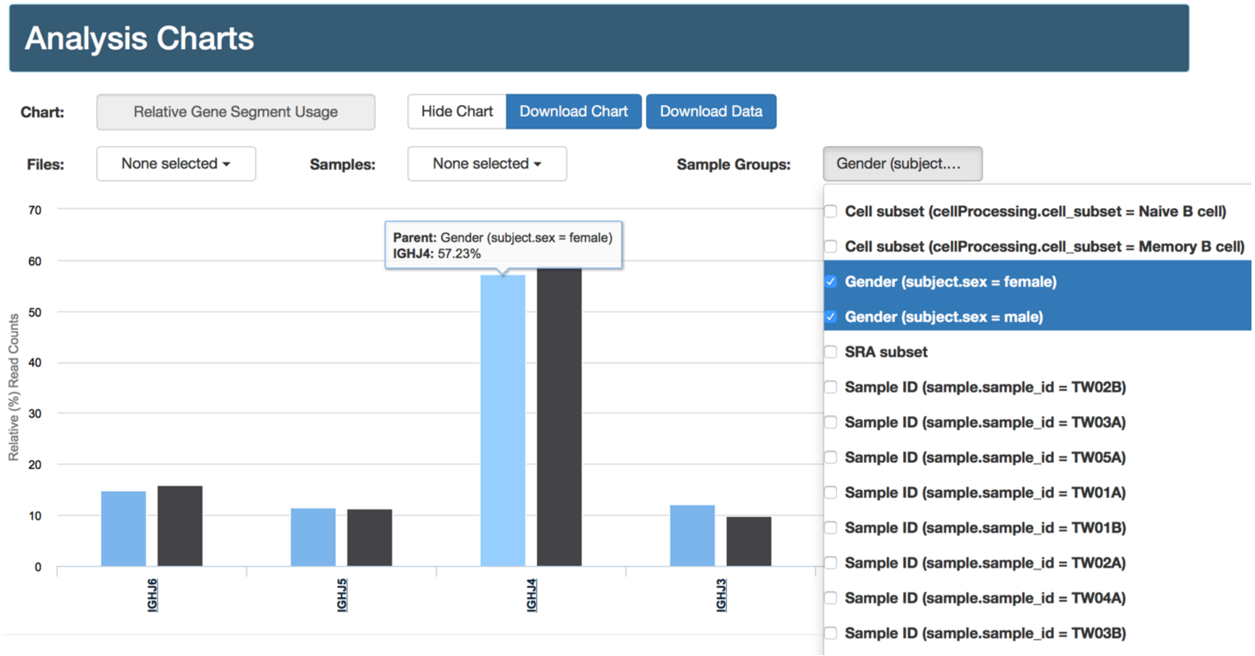

Supplementary Figure 24. RepCalc analysis chart for relative gene segment usage. Three popup lists are provided for Files, Samples, and Sample Groups, respectively. In the figure, the Sample Groups popup has been selected. Multiple selections can be performed and will be displayed together on the chart. In this figure, two sample groups are selected, female (blue bars) and male (black bars), for IGHJ gene segments.
